# Analytical assessment of physical characteristics, metabolic processes, and molecular investigations of selected wheat (*Triticum* spp.) cultivars

**DOI:** 10.1186/s12870-025-07134-0

**Published:** 2025-08-12

**Authors:** Dina H. M. Shehata, Mohamed M. El-Mahdy, Mohamed Ibrahim, Magda M. I. EL Araby, Somia S. El -Akkad, Faten Y. Ellmouni

**Affiliations:** 1 Department of Botany, Faculty of Science, Ain Shams University, Cairo, Egypt; 2 Agricultural Genetic Engineering Research Institute (AGERI), Agricultural Research Centre (ARC), Giza, Egypt; 3 Botany Department, Faculty of Science, Fayoum University, Fayoum, 63514 Egypt

**Keywords:** Wheat, Grain quality, Physical characters, Nutritional value, Protein profiling, SCoT, *rbc*L chloroplast DNA barcoding

## Abstract

**Background:**

Wheat, a primary cereal crop, is crucial in addressing global food security. Understanding genetic diversity and conserving wheat germplasm is essential for developing cultivars resilient to climate change. This study investigates grain quality, nutritional profiles, and genetic diversity across a selection of Egyptian and internationally sourced wheat cultivars. Physical and chemical analyses were conducted to assess grain/flour quality, hardness, and micronutrient content. Genetic diversity was evaluated using protein profiling, SCoT markers, and *rbc*L chloroplast DNA barcoding, chosen for its highly conserved nature and proven utility in plant species identification and phylogenetic analysis, making it a reliable marker for assessing genetic relationships among wheat cultivars. The findings from this study revealed distinct patterns of genetic variation and highlight valuable traits within the germplasm, providing crucial information for developing wheat cultivars adapted to diverse climatic conditions.

**Results:**

Physical and biochemical analyses revealed that two Egyptian cultivars, Sohag 5 and Misr 1, exhibited superior quality and nutritional value among the nine evaluated wheat cultivars. Both showed favorable physical properties (e.g., grain weight, falling number, gluten content). Sohag 5 was notably rich in carbohydrates, protein content, and essential minerals (zinc, calcium, magnesium), while Misr 1 also maintained healthy carbohydrate and gluten levels. Genetic diversity analysis, employing SDS-PAGE protein profiling and SCoT markers, effectively differentiated the wheat cultivars. These molecular markers consistently grouped the cultivars, generally distinguished between bread wheat and durum wheat varieties, and provided insights into the genetic relationships between Egyptian and imported lines. While the specific clustering patterns varied between marker types, particularly with *rbc*L sequences providing a distinct grouping since the *rbc*L chloroplast gene exhibited limited resolution for differentiating closely related cultivars. The combined genetic data confirmed significant diversity within the germplasm. Overall, the analysis identified two primary genetic groups among the cultivars, with Group I comprising seven diverse cultivars and Group II containing two distinct cultivars (Benisuif 6 and Sohag 5).

**Conclusions:**

Overall, the investigated Egyptian wheat cultivars demonstrated competitive or superior performance in standard physical and nutritional parameters compared to the imported varieties, with Sohag 5 and Misr 1 notably excelling in grain quality and micronutrient content. The genetic diversity analysis, incorporating protein profiling, SCoT markers, and *rbc*L chloroplast DNA barcoding, effectively characterized the genetic landscape of the cultivars. A key finding was the consistent genetic distinction of specific Egyptian cultivars, notably Sohag 5 and Benisuif 6, which clustered uniquely, aligning with their classification as durum wheat varieties. This revealed genetic relationships, alongside the identified superior traits (e.g., in Sohag 5), provides valuable insights that can be strategically utilized in breeding programs to develop new wheat cultivars with enhanced quality and adaptability to diverse climatic conditions.

**Supplementary Information:**

The online version contains supplementary material available at 10.1186/s12870-025-07134-0.

## Background

Wheat (*Triticum aestivum* L.), a cornerstone of human diet, offers a comprehensive nutrient profile. Comprising approximately 55% carbohydrates, 14% protein, and essential vitamins and minerals such as zinc, iron, phosphorus, magnesium, thiamine, niacin, and vitamin B6, wheat provides a balanced nutritional foundation [1]. The grain’s structure, divided into bran, germ, and endosperm, further contributes to its nutritional diversity [2].

Egypt’s wheat production has seen advancements with the development of 16 high-yielding, pest-resistant varieties [3]. Despite these efforts, the country still relies on wheat imports to meet domestic demand. The National Wheat Research Program (NWRP) has played a pivotal role, introducing varieties like Gemmiza 7 and Giza 168, known for their superior performance [4].

Wheat grain quality, defined by parameters such as moisture content, falling number, and gluten content, is critical for successful milling, baking, and storage stability [5]. Foreign matter contamination can also adversely affect milling yield and flour quality [6]. Moisture content is fundamental as it impacts milling and storage. The falling number test assesses enzyme activity, which is crucial for bread quality and dough properties [7, 8]. Ash content, primarily from the bran, significantly influences milling yield and flour grade [7, 9, 10]. The protein content, particularly gluten proteins like gliadin and glutenin, is vital for dough characteristics and product suitability [4, 11]. Wet gluten content serves as a key indicator of protein levels for the food industry [7]. Beyond starch, wheat also provides essential minerals, B-complex vitamins, and beneficial bioactive components, with its mineral content often indicating flour grade [9, 10, 12].

Conserving and assessing wheat genetic resources are crucial for developing climate-resilient cultivars [13, 14]. All pre-breeding materials including cultivars are essential for marker-assisted selection for breeding programs to enhance wheat productivity [15]. SCoT (Start Codon Targeted) markers, based on the ATG translation start codon, have been widely used in cultivar characterization, genetic diversity assessment, and marker-assisted breeding [16–22]. DNA barcoding, particularly using the *rbc*L gene, has emerged as a valuable tool for plant identification and phylogenetic studies [23–28]. While some studies have explored the genetic diversity of wheat cultivars [29], a comprehensive understanding remains limited.

The nine selected cultivars in this study were chosen based on their adaptation to extreme climatic conditions and their potential to contribute to breeding programs [30, 31]. Preliminary analyses of grain characteristics, physical properties, and yield components further supported the selection of these cultivars. Additionally, some of these genotypes harbor abiotic stress resistance genes and transcription factors that have a potential impact in plant development and growth [30, 31].

The present study focused on investigating grain quality, nutritional profiles of nine wheat cultivars from diverse origins using a combination of physical and biochemical analyses. Furthermore, assessing the genetic variability by employing molecular markers of different types was also addressed. The molecular markers included, SCoT and *rbc*L chloroplast DNA barcoding gene. The findings of this study will contribute to the development of improved wheat cultivars, particularly in the context of the global goal of doubling wheat yield by 2050 [15].

## Results

### Physical analyses

**Fig. 1:**
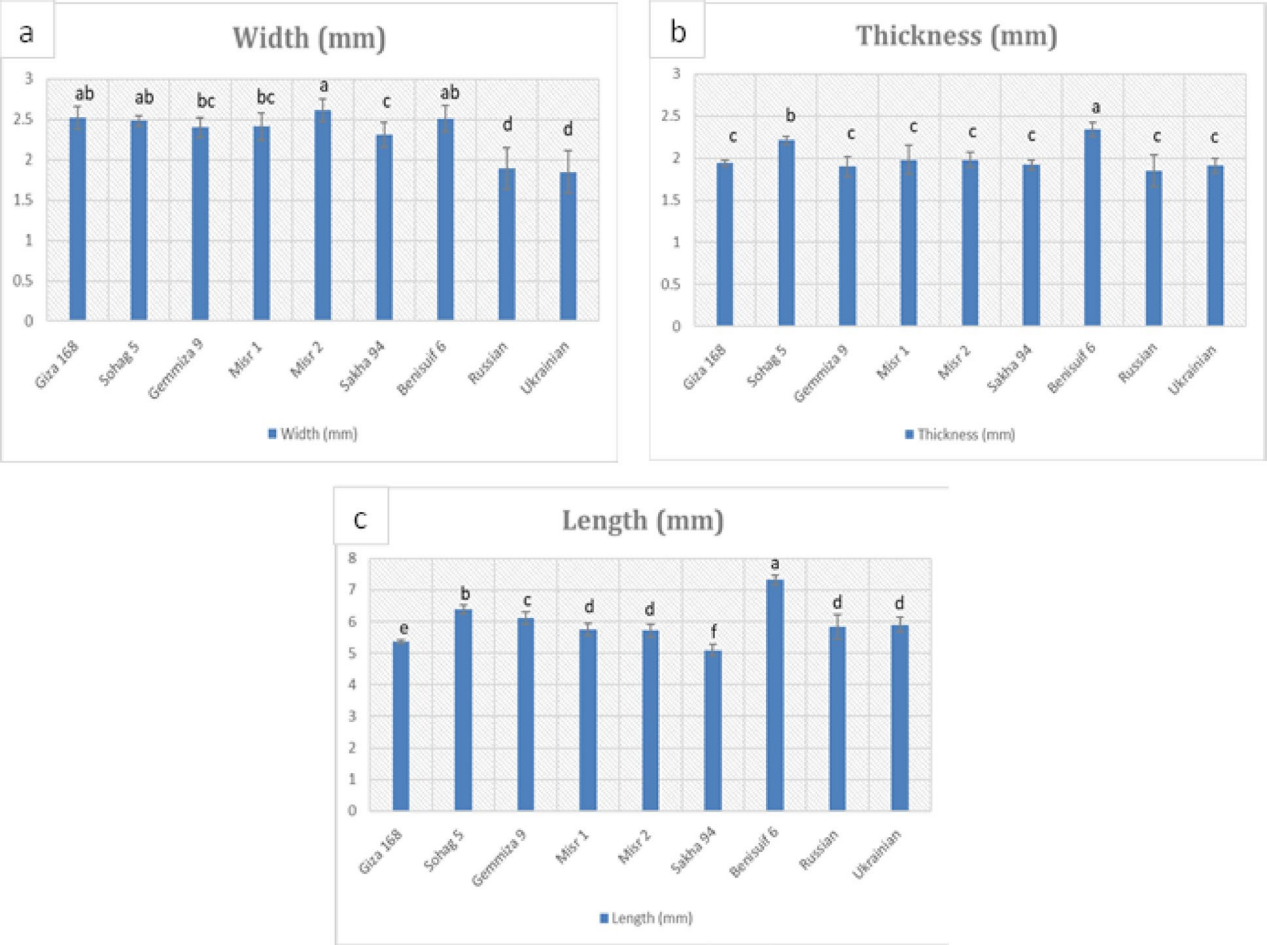
Grain dimensions (width, thickness, and length) of nine wheat cultivars in millimeters (mm). The cultivars include seven Egyptian cultivars (Benisuif 6, Gemmiza 9, Giza 168, Misr 1, Misr 2, Sakha 94, and Sohag 5) and two foreign cultivars (Russian and Ukrainian). Vertical bars represent ± SD

**Fig. 2:**
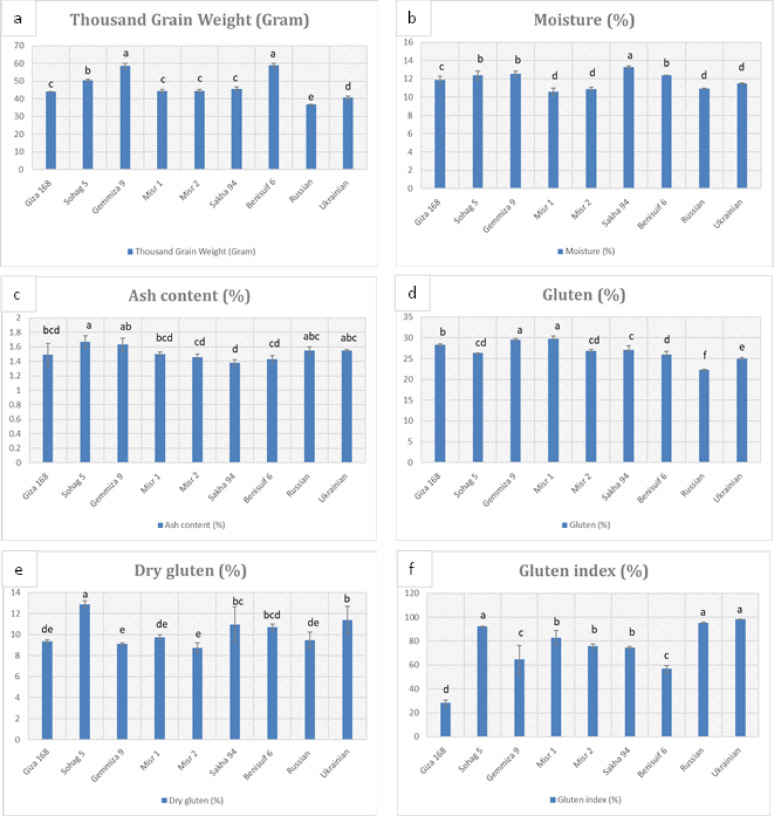
Physical analyses of grains and whole wheat flour of nine wheat cultivars: (a) thousand grain weight (for grains), (b) moisture content (for whole wheat flour), (c) ash content (for whole wheat flour), d) Gluten (for whole wheat flour), e) Dry gluten (for whole wheat flour) and f) Gluten index (for whole wheat flour). The cultivars include seven Egyptian cultivars (Benisuif 6, Gemmiza 9, Giza 168, Misr 1, Misr 2, Sakha 94, and Sohag 5) and two foreign cultivars (Russian and Ukrainian). Vertical bars represent ± SD

Grain dimension analysis performed on the studied wheat cultivars (Fig. 1, Fig. S1, Table S1) revealed significantly smaller grain sizes in foreign cultivars compared to Egyptian cultivars. Durum wheat cultivars, Benisuif 6 and Sohag 5, exhibited the greatest thickness (2.34 mm and 2.21 mm, respectively) and the longest grain lengths (7.32 mm and 6.40 mm, respectively). Thousand-grain weight analysis (Fig. 2a, Table S2) showed that foreign wheat cultivars, Russian and Ukrainian, had the lowest weights (36.9 g and 40.57 g, respectively). In contrast, Benisuif 6 and Gemmiza 9 displayed the highest thousand-grain weights.

The moisture content of whole wheat flour ranged from 10.6 to 13.30% (Fig. 2b, Table S2), with Sakha 94 having the highest moisture content. Misr 1, Misr 2, and Russian cultivars exhibited the lowest moisture levels. The ash content of whole wheat flour ranged from 1.38 to 1.67% (Fig. 2c, Table S2), with Sohag 5 having the highest value. The remaining cultivars showed relatively similar ash content.

The seven Egyptian wheat cultivars exhibited higher gluten content (26-29.8%) compared to the foreign cultivars (22.30-24.97%) (Fig. 2d, Table S2). Misr 2 and Gemmiza 9 had lower dry gluten content, while Sohag 5 and Ukrainian showed higher values (Fig. 2e, Table S2). Giza 168 and Benisuif 6 had lower gluten index values, while Ukrainian, Russian, and Sohag 5 exhibited higher values (Fig. 2f, Table S2).

**Fig. 3:**
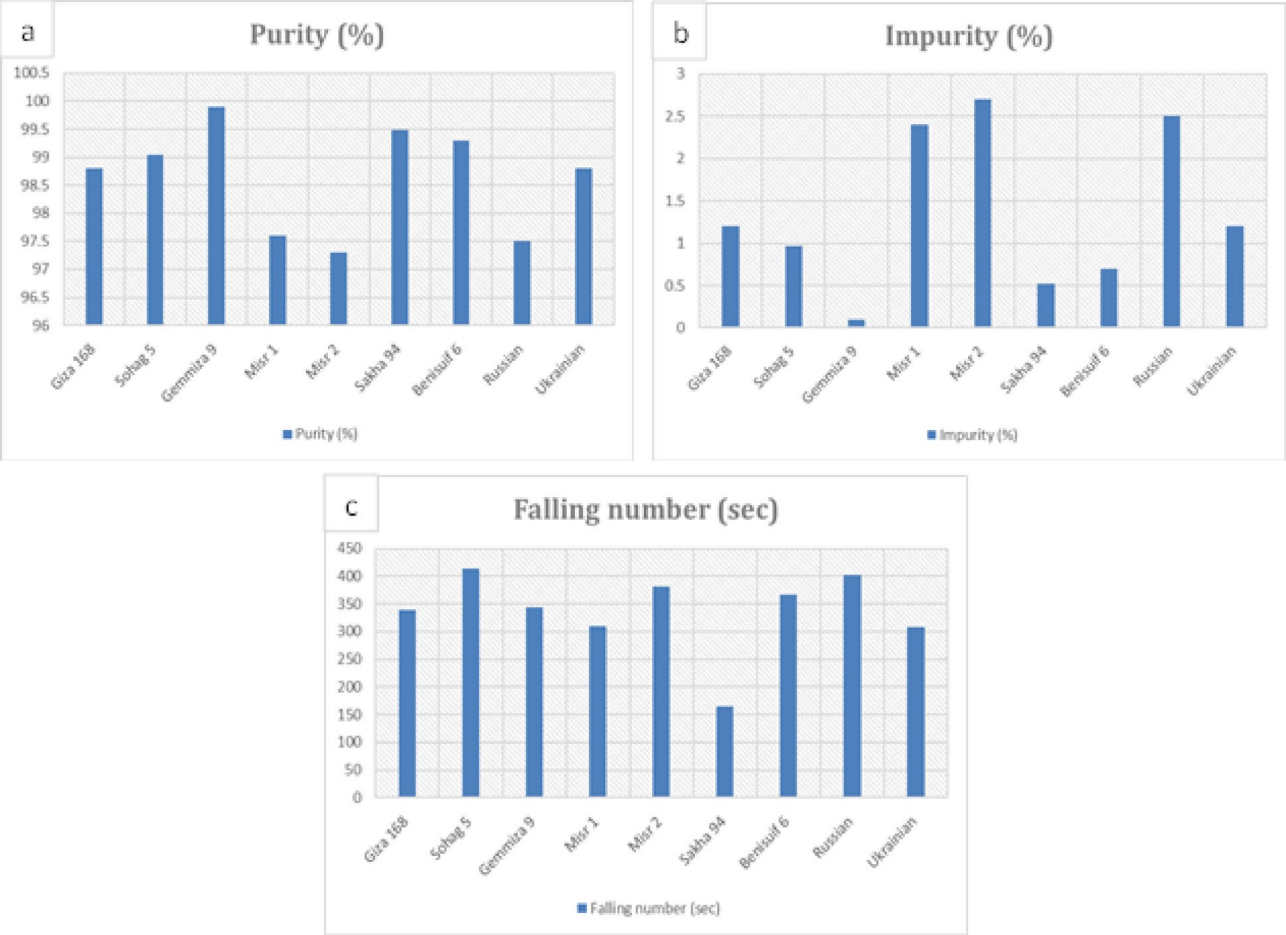
Physical analyses of grains and whole wheat flour of nine wheat cultivars: (a) purity percentage (for grains), (b) impurity percentage (for grains), and (c) fallen number (for whole wheat flour). The cultivars include seven Egyptian cultivars (Benisuif 6, Gemmiza 9, Giza 168, Misr 1, Misr 2, Sakha 94, and Sohag 5) and two foreign cultivars (Russian and Ukrainian)

All cultivars had a purity level above 97%, with Gemmiza 9 showing the highest purity (99.90%) and Misr 2, Misr 1, and Russian having slightly higher impurity content (Fig. 3a, b, Table S3). Sakha 94 had the lowest falling number (165 s), while Sohag 5 had the highest (413 s) (Fig. 3c, Table S3). The remaining cultivars showed relatively similar falling number values.

### Biochemical analyses

**Fig. 4:**
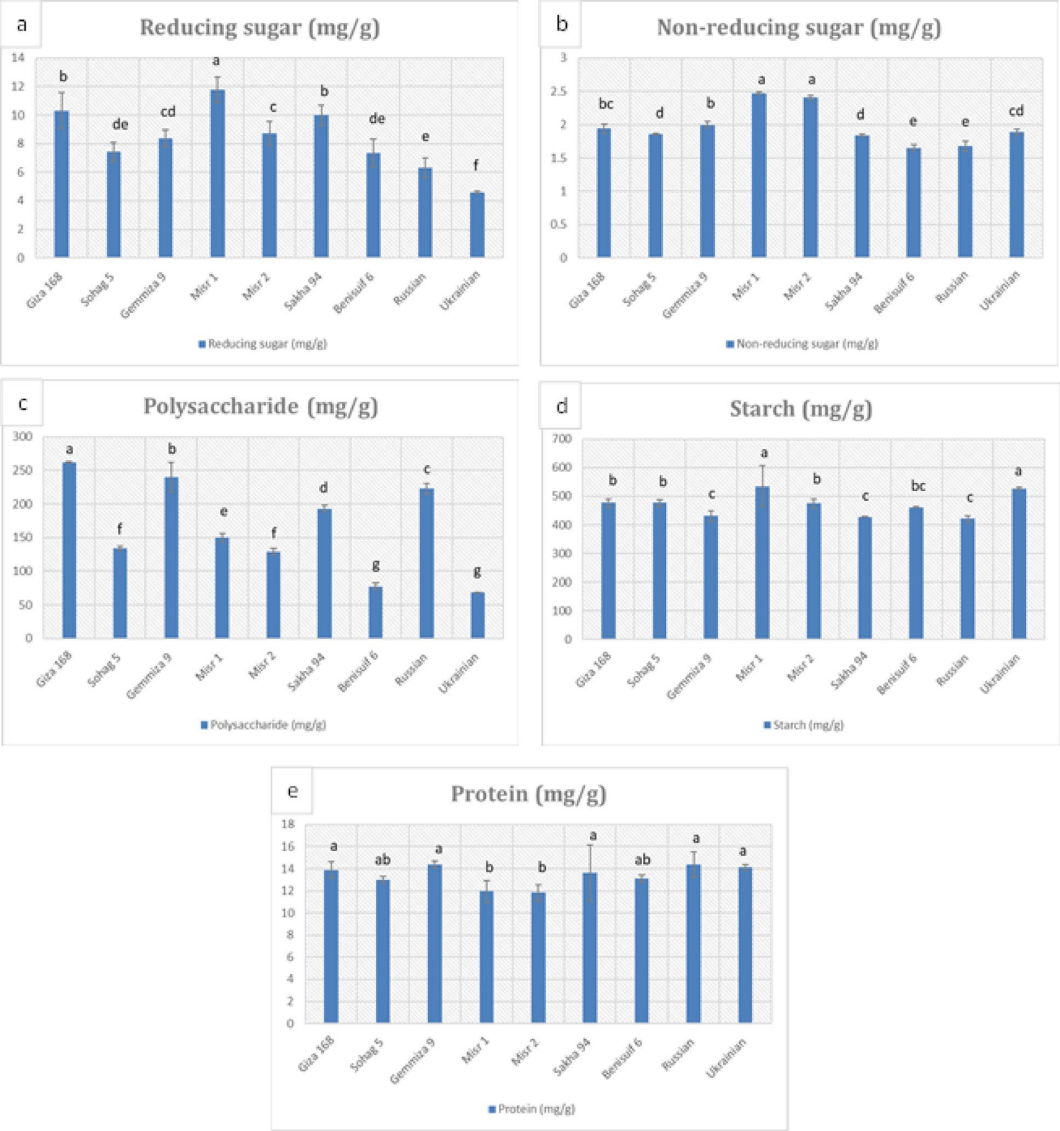
Total carbohydrate content (reducing sugars, non-reducing sugars, polysaccharide and starch) and total soluble protein of whole wheat flour of nine wheat cultivars. The cultivars include seven Egyptian cultivars (Benisuif 6, Gemmiza 9, Giza 168, Misr 1, Misr 2, Sakha 94, and Sohag 5) and two foreign cultivars (Russian and Ukrainian). Vertical bars represent ± SD

Whole wheat flour from Giza 168 exhibited the highest total carbohydrate content (751.19 mg/g), while Benisuif 6 had the lowest (548.6 mg/g) (Fig. 4, Table S4). Misr 1, Giza 168, and Sakha 94 had the highest reducing sugar content, while Misr 1 and Misr 2 had the highest non-reducing sugar content (Fig. 4a, b, Table S4). Ukrainian and Benisuif 6 had the lowest polysaccharide content, while Giza 168 and Gemmiza 9 had the highest (Fig. 4c, Table S4). Misr 1 and Ukrainian had the highest starch content, while the other cultivars had similar levels (Fig. 4d, Table S4).

**Fig. 5:**
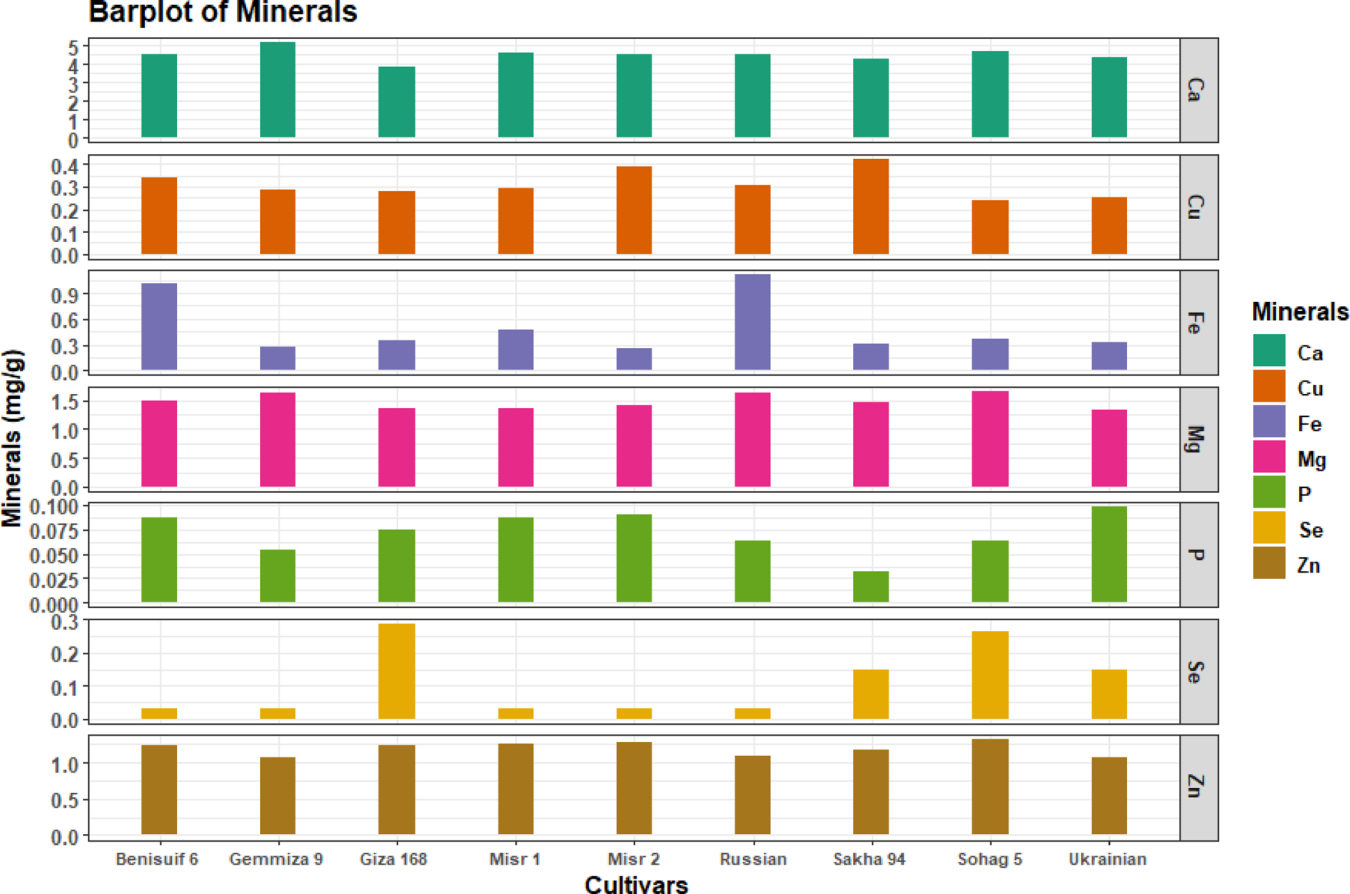
Minerals content (mg/g) of wheat whole flour of nine wheat cultivars is presented in a barplot chart. The cultivars included are Benisuif 6, Gemmiza 9, Giza 168, Misr 1, Misr 2, Russian, Sakha 94, Sohag 5, and Ukrainian

Total soluble protein content ranged from 11.83 mg/g to 14.40 mg/g, with Misr 2 and Misr 1 having the lowest levels (Fig. 4e, Table S4). All cultivars exhibited high calcium content and low levels of phosphorus and selenium (Fig. 5, Table S5). Sohag 5 had the highest magnesium and zinc content, while Sakha 94 had the highest copper content. Russian wheat had the highest iron content, and Gemmiza 9 had the highest calcium content.

**Fig. 6:**
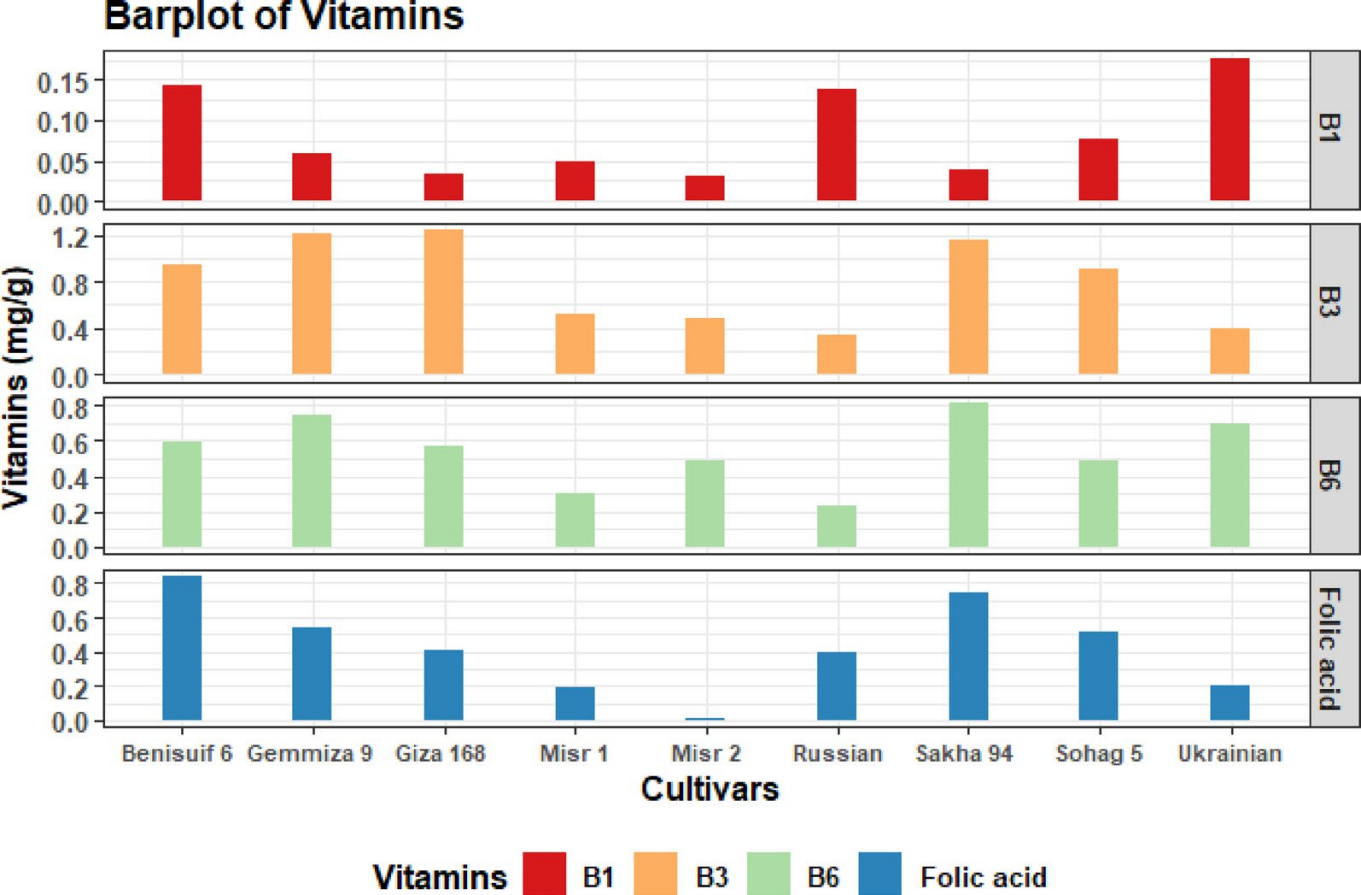
Vitamins content (mg/g) of wheat whole flour of nine wheat cultivars is presented in a barplot chart. The cultivars included are Benisuif 6, Gemmiza 9, Giza 168, Misr 1, Misr 2, Russian, Sakha 94, Sohag 5, and Ukrainian

All cultivars had high levels of niacin, low levels of thiamine, and moderate levels of pyridoxine and folic acid (Fig. 6, Table S6). Ukrainian wheat had the highest thiamine content, followed by Benisuif 6. Misr 2 had the lowest thiamine content. Giza 168 had the highest niacin content, and Sakha 94 had the highest pyridoxine content. Benisuif 6 and Sakha 94 had the highest folic acid content.

**Fig. 7:**
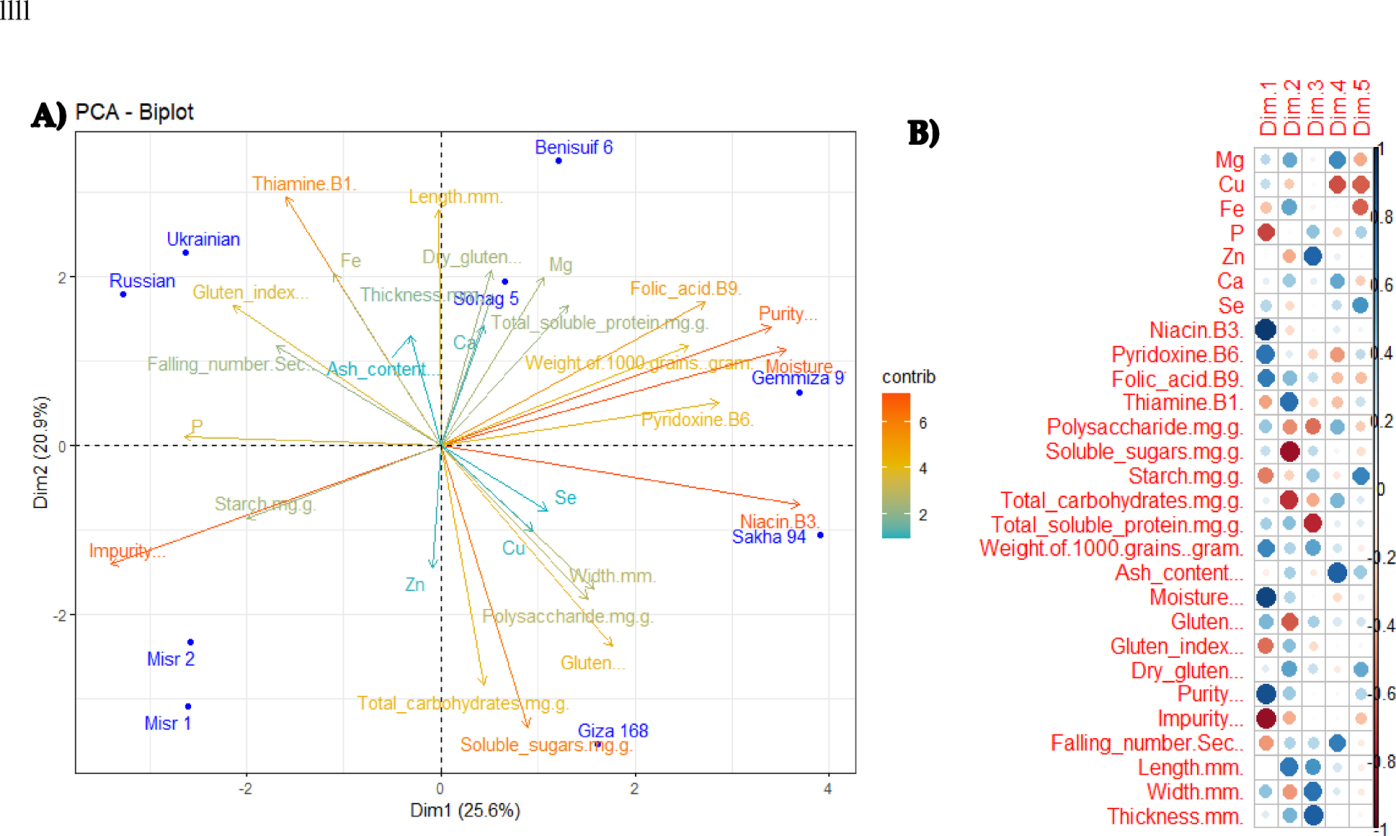
(A) Principal Component Analysis (PCA) of nine wheat cultivars (Benisuif 6, Gemmiza 9, Giza 168, Misr 1, Misr 2, Russian, Sakha 94, Sohag 5 and Ukrainian) based on 28 quantitative characteristics. (B) Correlation between different analyzed variables

Principal Component Analysis (PCA) of nine wheat cultivars (Benisuif 6, Gemmiza 9, Giza 168, Misr 1, Misr 2, Russian, Sakha 94, Sohag 5, and Ukrainian) based on 28 quantitative characteristics (as revealed by biophysical and biochemical analyzed parametsers) was generated with a total of 25.6% described along the first axis (Dim 1) and a total of 20.9% described along the second axis (Dim 2) (Fig. 7a). Furthermore, the correlation between different variables was addressed (Fig. 7B), whereas niacin (B3) and thiamine (B1) contributed the most to dimension 1 and dimension 2, respectively. These results highlighted the importance of purity, moisture, soluble sugars, and vitamins (specifically B1 and B3) measurements as an indispensable key parameters (since they have longest rays originated from the centre) in the differentiation of studied wheat cultivars (Fig. 7a, b).

### Molecular characterization and genetic relationships

#### Molecular characterization and genetic relationships as revealed by SDS-PAGE technique

To assess the genetic diversity among the nine wheat cultivars (Giza 168, Sohag 5, Misr 1, Misr 2, Sakha 94, Benisuif 6, Russian, and Ukrainian), SDS-PAGE analysis was employed to examine their protein profiles. Total proteins were extracted from milled wheat grains and separated using Laemmli’s method [32].

**Fig. 8:**
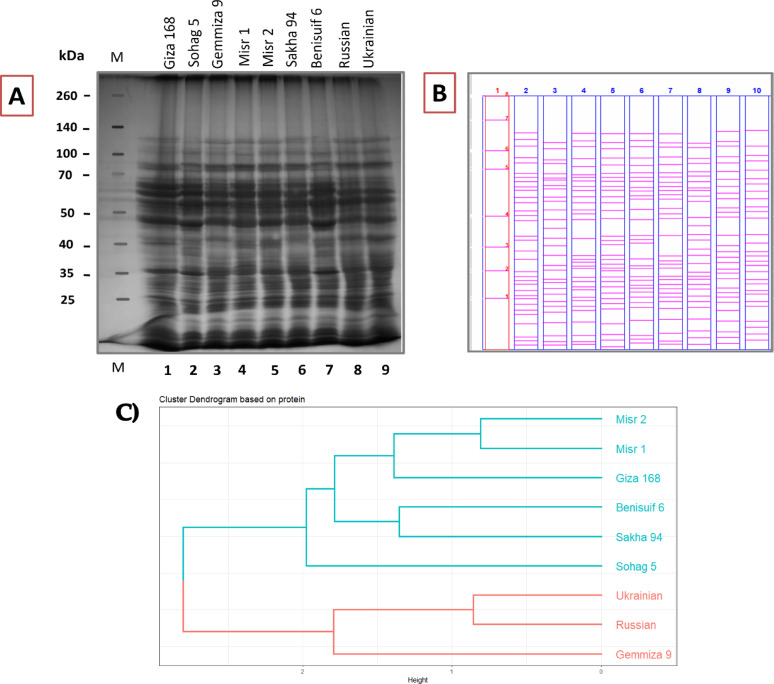
Electrophoretic banding patterns of extracted total cellular proteins (TCPs) of nine wheat cultivars using (A) SDS-PAGE technique shown by (B) schematic representation. Lane M: protein marker, Lane 1: Giza 168, Lane 2: Sohag 5, Lane 3: Gemmiza 9, Lane 4: Misr 1, Lane 5: Misr 2, Lane 6: Sakha 94, Lane 7: Benisuif 6, Lane 8: Russian, Lane 9: Ukrainian. (C) Cluster analysis was performed based on the SDS-PAGE fractionated total proteins (TPs) of the nine examined samples of wheat cultivars (seven Egyptian cultivars; Benisuif 6, Gemmiza 9, Giza 168, Misr 1, Misr 2, Sakha 94, and Sohag 5 and two foreign cultivars; Russian and Ukrainian). A dendrogram was constructed using scored data from the fractionated TPs in the R program

Protein profiles of nine wheat cultivars were visualized using linear representations which were generated from band scoring analysis to identify protein variations (Fig. 8a, b). Band scoring, assessing protein band presence/absence, was conducted using PyElph 1.4 and Image Lab software (Table S7). A total of 44 protein bands were detected, revealing 77.27% polymorphism, indicating substantial protein diversity. Eight unique bands and ten monomorphic ones were identified, with 34 polypeptides exhibiting polymorphism (Table S7).

A “Negative Unique Band” (NUB) is defined by its absence in one cultivar and presence in all others. These findings suggest highly specific protein characteristics among the studied cultivars.

Cluster analysis (Fig. 8c) revealed two major clusters. Cluster I encompassed six Egyptian cultivars (Benisuif 6, Sakha 94, Misr 2, Misr 1, Giza 168, and Sohag 5), further divided into two subclusters: (1) Misr 2, Misr 1, and Giza 168; and (2) Benisuif 6 and Sakha 94. Sohag 5 formed a separate branch within Cluster I. Cluster II comprised three cultivars: Gemmiza 9 (single branch) and a subcluster containing Ukrainian and Russian wheat cultivars.

#### Molecular characterization and genetic relationships as revealed by scot markers

**Fig. 9:**
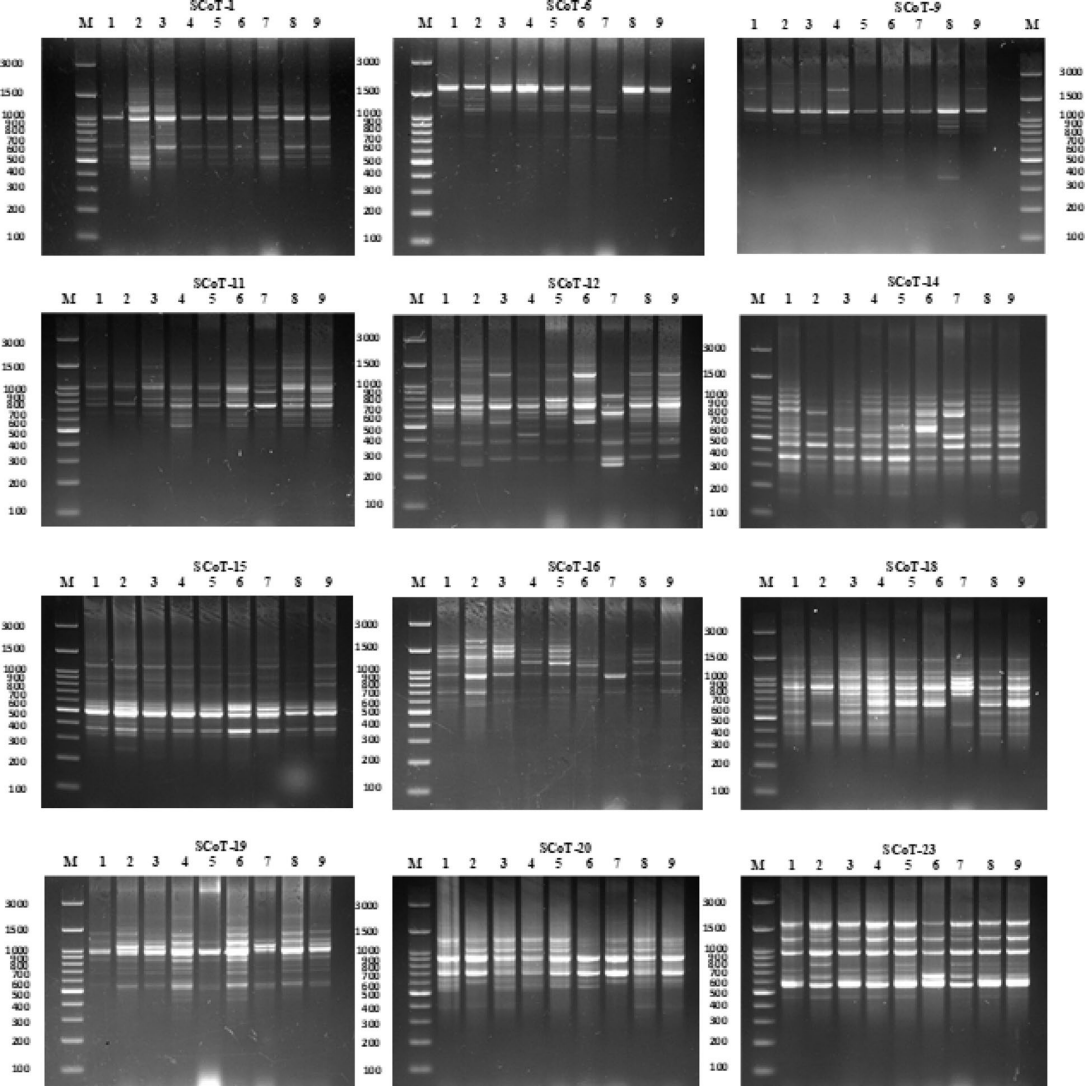
Photographs of representative agarose gel electrophoresis of PCR amplicons of SCoT primers. Comparison of the twelve SCoT-PCR profiles of the nine wheat cultivars under study. M: 100 bp DNA size marker. Lane order: 1–9 (left to right); Lane 1: Giza 168, Lane 2: Sohag 5, Lane 3: Gemmiza 9, Lane 4: Misr 1, Lane 5: Misr 2, Lane 6: Sakha 94, Lane 7: Benisuif 6, Lane 8: Russian, and Lane 9: Ukrainian

**Fig. 10:**
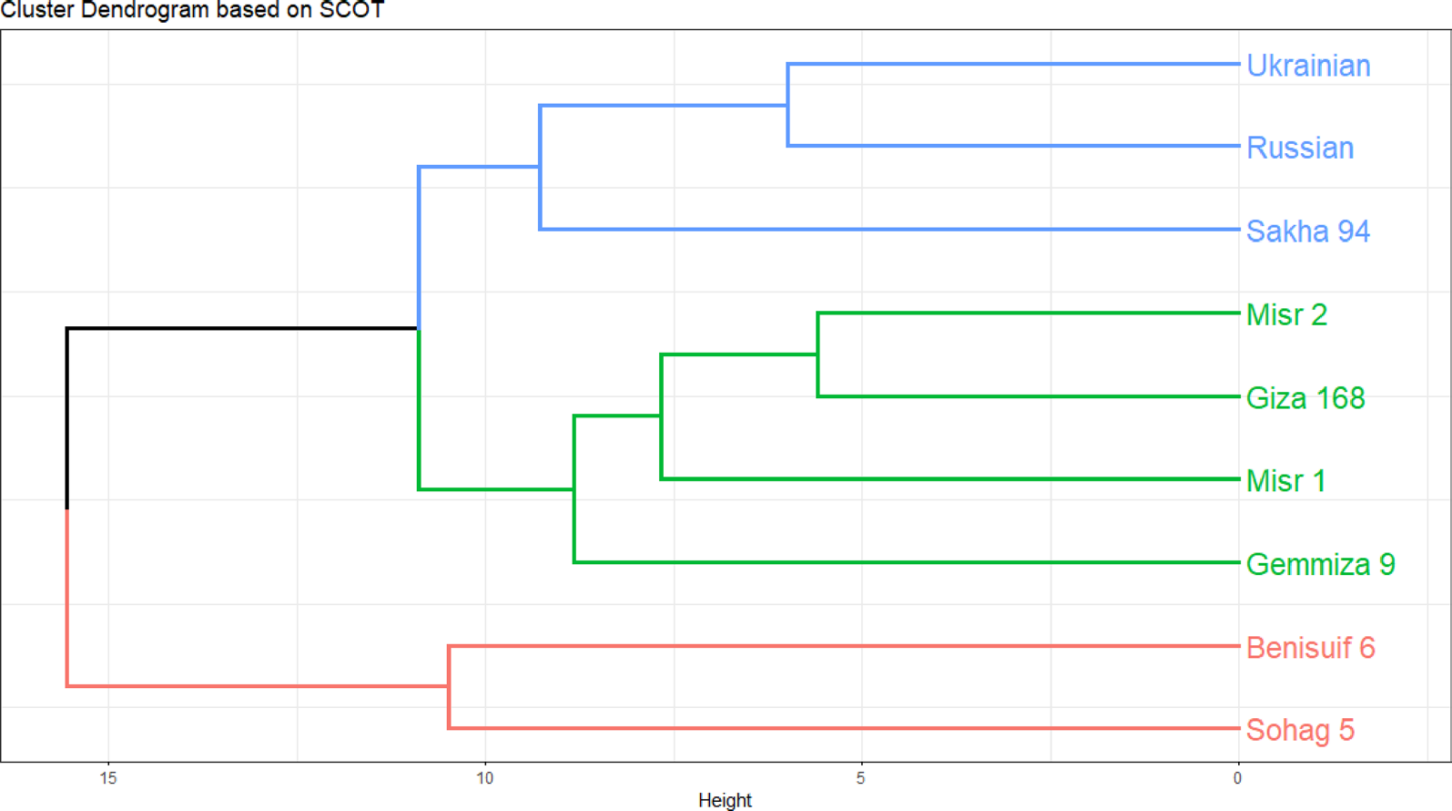
Dendrogram based on the SCOT marker for the nine wheat cultivars (seven Egyptian cultivars; Benisuif 6, Gemmiza 9, Giza 168, Misr 1, Misr 2, Sakha 94 and Sohag 5, and two foreign cultivars; Russian and Ukrainian)

Twelve SCoT primers were employed for polymorphism analysis, generating 148 amplicons, of which 105 (70.96%) were polymorphic. Polymorphism percentage ranged from 55.56% (Primer SCoT 20) to 88.89% (Primer SCoT 6). Both Primer SCoT 6 and Primer SCoT 20 yielded nine scorable bands. The average Polymorphism Information Content (PIC) was 0.26, ranging from 0.2 (Primer SCoT 15) to 0.36 (Primer SCoT 9). The average Effective Multiplex Ratio (EMR) was 0.086, varying from 0.048 (Primer SCoT 12) to 0.125 (Primer SCoT 9). Finally, the average Marker Index (MI) was 2.31, with a range of 1.12 (Primer SCoT 20) to 2.92 (Primer SCoT 1) (Table 1; Fig. 9). The cluster analysis raised Misr 2, Giza 168, and Misr 1, with Gemmiza 9 forming a separate branch. The second subcluster confirmed the grouping of Ukrainian and Russian wheat, along with a single branch for Sakha 94. All cultivars in group I were bread wheat. Cluster II consisted solely of durum cultivars; Benisuif 6 and Sohag 5 (Fig. 10).

**Table 1:** The list of scot primers name, sequence, GC%, total number of amplicons (TNAs), monomorphic amplicons (MAs), polymorphic amplicons (PAs), percentage of polymorphism (%P), polymorphism information content (PIC), effective multiplex ratio (EMR), and marker index (MI) as revealed by scot polymorphism marker analysis in 9 wheat cultivars. The primer sequences were synthesized by the HVD Egypt company

Serial no.	Primer Name	Sequence (5‘ › 3‘)	GC%	TNAs	MAs	PAs	% *P*	PIC	EMR	MI
1	SCoT 1	CAACAATGGCTACCACCA	50%	13	3	10	76.92	0.292	0.077	2.92
2	SCoT 6	CATGGCTACCACCGGCCC	72.2%	9	1	8	88.89	0.241	0.110	1.93
3	SCoT 9	CAACAATGGCTACCAGCA	50%	8	1	7	87.50	0.364	0.125	2.55
4	SCoT 11	AAGCAATGGCTACCACCA	50%	12	5	7	58.33	0.222	0.083	1.56
5	SCoT 12	ACGACATGGCGACCAACG	61.1%	21	4	17	80.95	0.313	0.048	5.32
6	SCoT 14	ACGACATGGCGACCACGC	66.7%	13	4	9	69.23	0.255	0.077	2.29
7	SCoT 15	ACGACATGGCGACCGCGA	66.7%	13	4	9	69.23	0.201	0.077	1.81
8	SCoT 16	ACCATGGCTACCACCGAC	61.1%	11	3	8	72.73	0.296	0.091	2.37
9	SCoT 18	ACCATGGCTACCACCGCC	66.7%	14	5	9	64.29	0.257	0.071	2.32
10	SCoT 19	ACCATGGCTACCACCGGC	66.7%	14	5	9	64.29	0.219	0.071	1.97
11	SCoT 20	ACCATGGCTACCACCGCG	66.7%	9	4	5	55.56	0.225	0.110	1.12
12	SCoT 23	CACCATGGCTACCACCAG	61.1%	11	4	7	63.64	0.229	0.091	1.60
Total				**148**	**43**	**105**	**-**	**-**	**-**	**-**
Mean				**-**	**-**	**-**	**70.96**	**0.260**	**0.086**	**2.31**

#### Molecular relationships of wheat cultivars based on protein profiling and scot markers

**Fig. 11:**
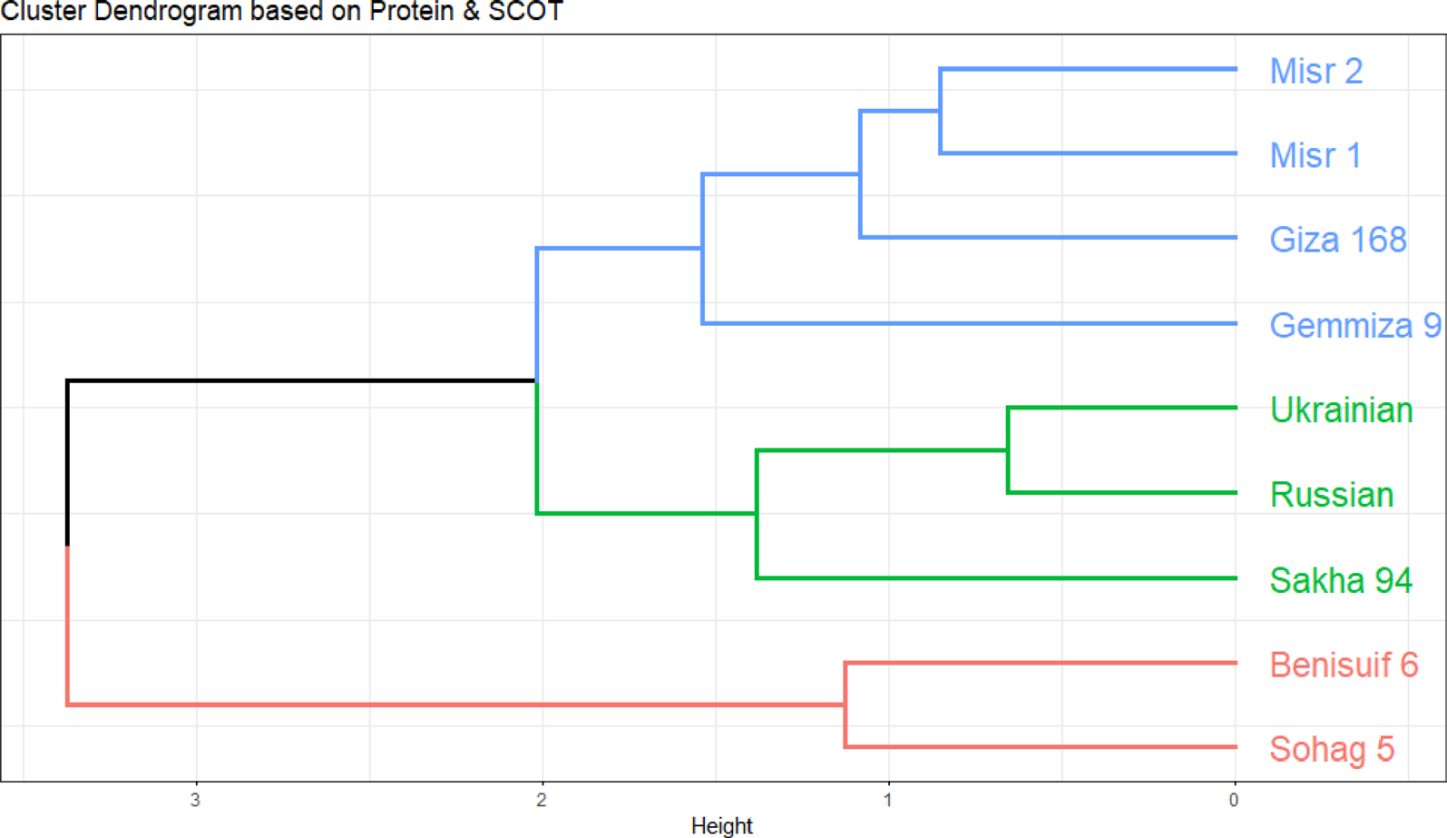
Cluster dendrogram visualizes the relationships among nine wheat cultivars (seven Egyptian cultivars; Benisuif 6, Gemmiza 9, Giza 168, Misr 1, Misr 2, Sakha 94 and Sohag 5, and two foreign cultivars; Russian and Ukrainian) based on (**A**) Protein patterns only, (**B**) SCoT markers only, and (**C**) Combined binary data of protein patterns and SCoT markers

Euclidean distance-based clustering was performed using combined binary data of protein patterns and SCoT markers for the nine wheat cultivars. Individual cluster analyses for protein patterns (Fig. 8c) and SCoT markers (Fig. 10) were also conducted. The dendrogram generated from the combined analysis (Fig. 11) largely mirrored the clustering pattern observed with SCoT markers. However, a notable difference emerged: Misr 1 and Misr 2 clustered together in the combined analysis, aligning with their grouping based on protein pattern data.

#### Relationship of wheat cultivars based on RbcL barcoding

**Fig. 12:**
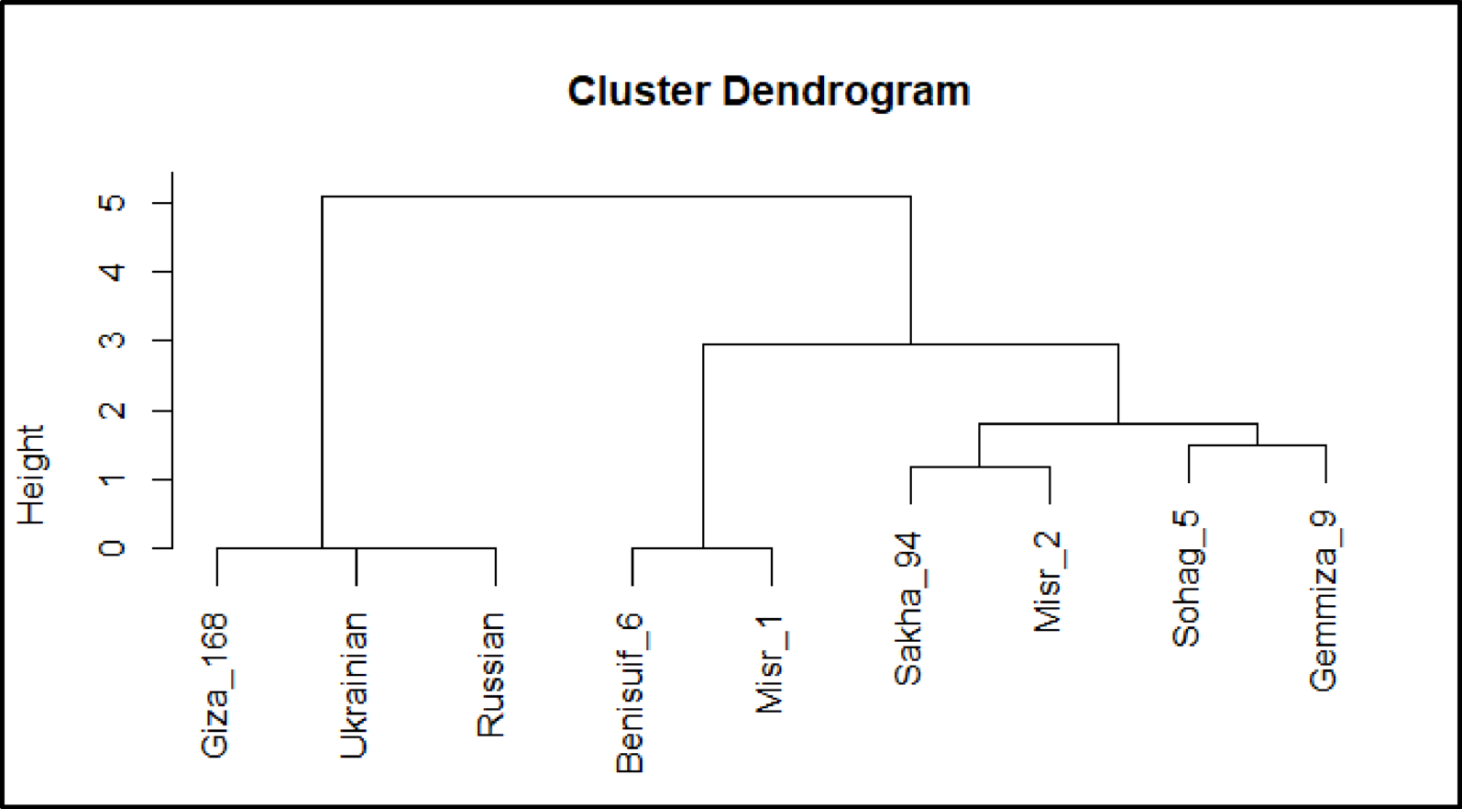
Cluster tree constructed using R software illustrating genetic relatedness among the nine wheat cultivars as revealed by sequence variation of *rbc*L barcodes

Amplification using *rbc*L gene-specific primers (Table 2) yielded DNA fragments of approximately 600 bp (Table 2, Fig. S2). BLASTN analysis confirmed that all sequences belonged to *T. aestivum* and *T. durum* by matching with NCBI GenBank entries for these species. Full *rbc*L chromatograms and sequences are provided as supplementary material. GenBank accession numbers for the obtained *rbc*L sequences are listed in Table 3. Sequence variations-based phylogenetic tree of *rbc*L gene (Fig. 12) illustrated the genetic relationships among the nine studied genotypes. This analysis revealed two distinct groups. Group I comprised Giza 168, Russian, and Ukrainian cultivars, while the remaining cultivars formed Group II.

**Table 2:** Primer codes and sequences for barcoding of the *rbc*L gene and their size in bp

Primer Code	Sequence	Product Size	Reference
*rbc*La-1 F	5’-ATGTCACCACAAACAGAGACTAAAGC-3’	600 bp	Fay et al. (1997)
*rbc*L724-R	5’-TCGCATGTACCTGCAGTAGC-3’		

**Table 3:** Pedigree and accession *rbc*L number of nine wheat cultivars (seven Egyptian cultivars; Benisuif 6, gemmiza 9, Giza 168, Misr 1, Misr 2, Sakha 94 and Sohag 5, and two foreign cultivars; Russian (Bezostaya 1) and Ukrainian (Shestopalovka)

Serial No.	Taxa	Origin	Cultivar Name	Pedigree	Genebank Accession number of rbcL barcodes^#^
1	*Triticum aestivum*	Egypt	Giza 168	MIL/BUC//SERI	PP297465.1
2	*Triticum turgidum* subsp. *durum*	Egypt	Sohag 5	TRN//21,563/AA/3/BD2080/4/BD2339/5/Rascon37//Tarro 2//Rascon 3/6/Auk/Gull//Green	PP297466.1
3	*Triticum aestivum*	Egypt	Gemmiza 9	ALD’S’/HUAC’S’//CMH74.630/5X	PP297467.1
4	*Triticum aestivum*	Egypt	Misr 1	OASIS/SKAUZ//4*BCN/3/2*PASTOR	PP297468.1
5	*Triticum aestivum*	Egypt	Misr 2	SKAUZ/BAV 92	PP297469.1
6	*Triticum aestivum*	Egypt	Sakha 94	OPATA/RAYON/3/JUP/BJY//URES	PP297470.1
7	*Triticum turgidum* subsp. *durum*	Egypt	Benisuif 6	BOOMER-21/BUSCA-3	PP297471.1
8	*Triticum aestivum*	Russia	Russian (Bezostaya 1)	Bezostaya 4: Lutescens 17/Skorospelka 3*	PP297472.1
9	*Triticum aestivum*	Ukraine	Ukrainian (Shestopalovka)	N/A*	PP297473.1

*: Not applicable, ^#^: *rbc*L sequences were deposited under indicated accession numbers at the persistent web link approved by NCBI GenBank (https://www.ncbi.nlm.nih.gov/nuccore/).For example, PP297465.1 *T. aestivum rbc*L sequence could be retrieved using the following persistent web link (https://www.ncbi.nlm.nih.gov/nuccore/PP297465.1)

#### Molecular relationships between wheat cultivars based on protein profiling, scot and RbcL markers

**Fig. 13:**
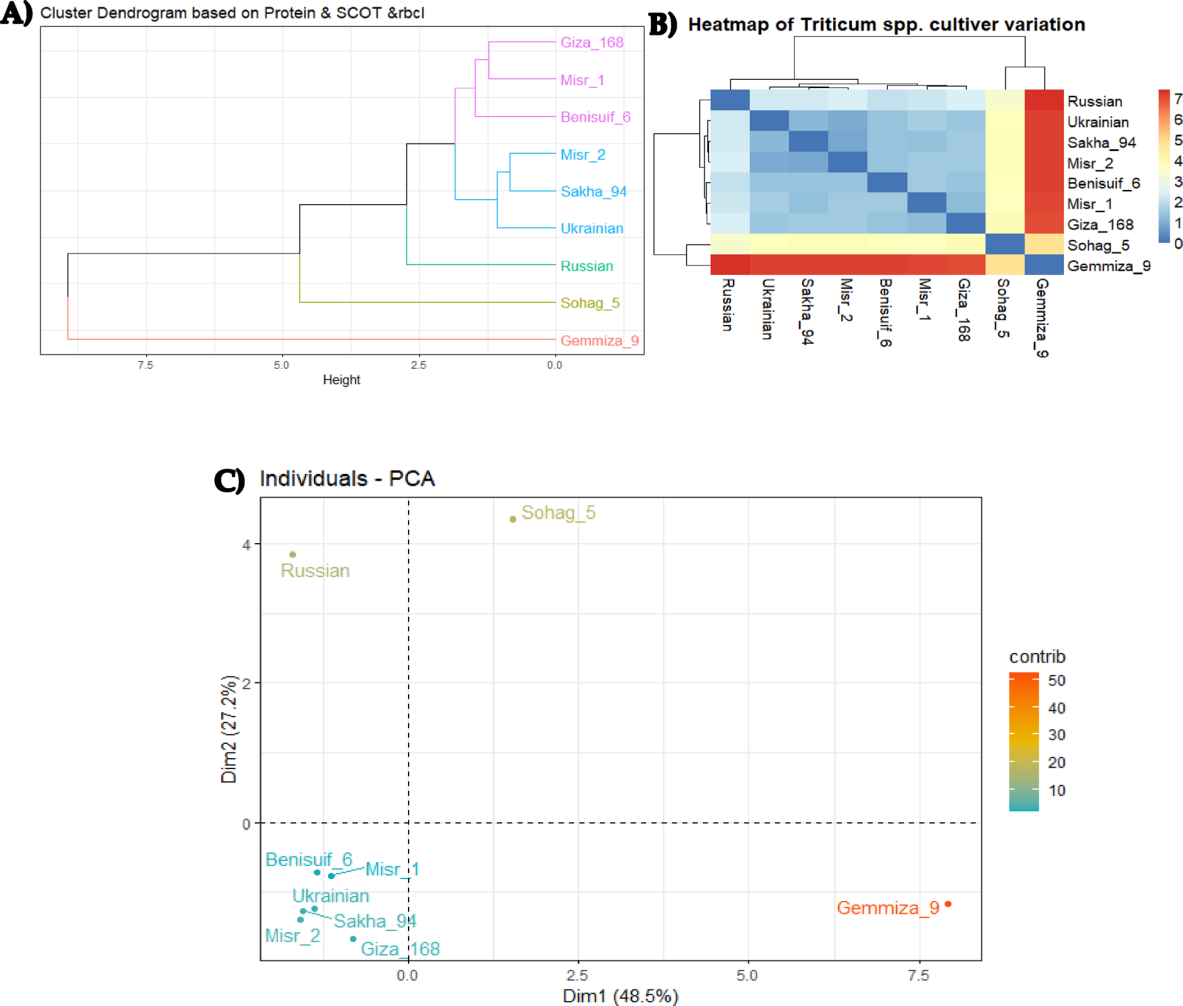
The results of combined data of protein, SCoT marker, and *rbc*L barcoding analyses for nine wheat cultivars (seven Egyptian cultivars; Benisuif 6, Gemmiza 9, Giza 168, Misr 1, Misr 2, Sakha 94 and Sohag 5, and two foreign cultivars; Russian and Ukrainian). **(A)** Cluster analysis, **(B)** Multivariate Heatmap, and **(C)** Principal Component Analysis (PCA) are used to analyze the relationships among the cultivars based on the employed molecular markers

Molecular relationships among wheat cultivars were investigated using three biostatistical analyses: cluster analysis, multivariate heatmap, and principal component analysis (PCA) (Fig. 13a, b, and c). These analyses consistently revealed distinct clustering patterns. Gemmiza 9, Sohag 5, and Russian cultivars consistently clustered separately. The remaining six cultivars formed two distinct groups: (1) Giza 168, Misr 1, and Benisuif 6; and (2) Misr 2, Sakha 94, and Ukrainian wheat cultivars (Fig. 13a, b, and c).

## Discussion

Analysis of grain dimensions, including length, width, and thickness, revealed significant variations among the studied wheat cultivars. Durum wheat cultivars (Benisuif 6 and Sohag 5) exhibited thicker and longer grains compared to bread wheat cultivars. This aligns with previous findings by Sahin and Sumnu [33]. Foreign cultivars (Russian and Ukrainian) generally exhibited narrower grains, a characteristic often associated with higher yield potential in modern wheat cultivars [11]. Bread wheat cultivars (Sakha 94 and Giza 168) tended to be shorter, consistent with the typical characteristics of bread wheat [34]. Stereomicroscopic analysis was employed to capture detailed images of wheat grain morphology. This approach, similar to that used by Liu et al. [35], allows for the analysis of grain shape and size variations. Microscopic analysis of grain surface characteristics has proven valuable for cultivar identification and differentiation [36]. While previous studies have utilized scanning electron microscopy (SEM) for such analyses [37], stereomicroscopy offers a practical and efficient method for assessing dimensional features relevant to breeding programs and quality assessment. Interestingly, Benisuif 6 and Gemmiza 9, both Egyptian cultivars, exhibited higher thousand-grain weights, aligning with the findings of Al-Mahturi [38]. This suggests that these cultivars may have potential for further breeding and improvement.

Variations in Thousand-Grain Weight (TGW) are pivotal for selecting wheat cultivars, as higher TGW is generally linked to plumper grains and increased flour yield [39]. However, TGW is frequently inversely associated with protein content, presenting a wellrecognized trade-off in breeding programs [40]. Given protein’s critical role in flour quality and dough strength, it is imperative for breeders to strategically balance the pursuit of high TGW with the necessity of maintaining adequate protein levels, thereby ensuring that overall flour quality is not compromised while optimizing yield potential.

The consistent and optimal moisture content observed across all wheat cultivars indicates effective postharvest handling and strong potential for long-term storage stability [41]. Maintaining such levels is crucial, as it minimizes the risk of fungal growth and insect infestation, thereby preserving overall grain quality and ensuring suitability for downstream processing.

Variations in ash content among cultivars have significant implications for milling and end product quality. Cultivars exhibiting higher ash content, such as Sohag 5, likely reflect elevated mineral levels [42], which, while nutritionally beneficial, may result in darker colored flour and baked goods [43]. Conversely, cultivars with lower ash content are generally preferred for superior milling efficiency and higher flour yield [44], highlighting a critical balance for commercial processing.

Egyptian wheat cultivars displayed higher gluten content compared to foreign cultivars, indicating strong bread-making potential [45]. Sohag 5 and Ukrainian cultivars exhibited high dry gluten content, potentially leading to higher flour yield [46]. Further investigation is needed to understand the genetic factors influencing gluten content variations within Egyptian cultivars. Foreign cultivars (Ukrainian and Russian) exhibited high gluten index values, reflecting strong gluten strength and elasticity. Encouragingly, the Egyptian cultivar Sohag 5 also showed high gluten index values, indicating the presence of valuable genetic resources for developing high gluten wheat cultivars. These findings align with previous studies [38].

High impurity levels in Misr 1, Misr 2, and Ukrainian cultivars can negatively impact the entire wheat value chain. Impurities provide ideal conditions for insect infestation and spoilage during storage, significantly reducing shelf life [6]. Additionally, foreign material can decrease market value and disrupt milling efficiency, leading to lower flour yields [47, 48].

All cultivars, except Sohag 5 and Sakha 94, exhibited FN values within the desirable range of 300–400s, indicating good inherent quality. Sohag 5’s high FN value (413 s) suggests minimal alphaamylase activity and suitability for hard bread baking [46]. However, Sakha 94’s significantly lower FN value (165 s) indicates potential pre-harvest sprouting damage and reduced bread-baking quality [1].

Analysis of carbohydrate content in wheat cultivars revealed significant variations, with Giza 168 exhibiting the highest (751.19 mg/g) and Benisuif 6 the lowest (548.6 mg/g) [49], suggesting genetic control over starch accumulation and cell wall composition. Reducing sugar content, influencing browning and sweetness during baking, was high in Misr 1, Giza 168, and Sakha 94, but lower in Benisuif 6, Ukrainian, and Russian cultivars [43]. Non-reducing sugars, important for yeast fermentation, were elevated in Misr 1 and 2, but lower in cultivars like Benisuif 6 and Russian [42]. Polysaccharide content (potentially impacting fiber) was lower in Ukrainian and Benisuif 6 compared to Giza 168 and Gemmiza 9 [44]. Misr 1 and Ukrainian also showed high starch content, a key energy source and determinant of flour yield.

Protein content is a key determinant of wheat’s economic value, given its paramount importance in bread-making and other food applications [6]. In this study, variations in total soluble protein were observed across cultivars, notably with some exhibiting lower values. This highlights the diverse genetic potential within the germplasm that can inform breeding programs to develop cultivars with targeted protein profiles. For example, the combination of high gluten content (as seen in Sohag 5) and a strong gluten index (characteristic of the Russian cultivar) is highly desirable for optimal bread wheat quality [1]. The complex interplay between gluten quantity, gluten strength, and overall soluble protein significantly influences crucial baking performance factors such as water absorption and dough viscosity [50, 51], thereby enabling the tailored development of wheat varieties for diverse food industry applications.

The studied wheat cultivars exhibited notable variations in their mineral content, highlighting distinct nutritional profiles within the germplasm. While calcium levels were generally robust across all cultivars, key minerals such as phosphorus and selenium were consistently present at low concentrations. Conversely, certain cultivars demonstrated enhanced accumulation of essential micronutrients; for instance, the Egyptian cultivar Sohag 5 was particularly rich in magnesium and zinc, while others like Sakha 94 and the Russian cultivar showed higher levels of copper and iron, respectively. These diverse mineral profiles are consistent with previous reports emphasizing the nutritional value of whole wheat flour [52]. The identification of these mineral rich cultivars holds significant implications for human health and breeding strategies aimed at biofortification, as the consumption of whole wheat provides vital dietary minerals crucial for a balanced diet and has been linked to positive cardiovascular health outcomes [50].

Regarding vitamins content, the nine cultivars displayed high niacin (vitamin B3) levels, with Giza 168 exhibiting the highest concentration (1.53 mg/g), followed by Gemmiza 9 (1.48 mg/g) and Sakha 94 (1.34 mg/g). This aligns with previous findings identifying wheat as a good source of niacin, an essential vitamin for energy metabolism and nervous system health [12]. The Ukrainian variety had the highest thiamine (vitamin B1) content (0.03 mg/g), followed by Benisuif 6 (0.02 mg/g), although wheat is generally considered a low source of this vitamin. Sakha 94 showed the highest pyridoxine (vitamin B6) content (0.65 mg/g). Finally, Sakha 94 and Benisuif 6 shared the highest folic acid content (0.7 and 0.56 mg/g, respectively) [12]. The high levels of B-complex vitamins, particularly niacin and folic acid, in these cultivars are significant, as they contribute substantially to human health by supporting metabolic processes, red blood cell formation, and preventing certain birth defects. This makes these cultivars particularly valuable for nutritional security and food fortification efforts [12].

In the other context, this study aimed to evaluate the genetic diversity among nine wheat cultivars, including seven Egyptian cultivars (Benisuif 6, Gemmiza 9, Giza 168, Misr 1, Misr 2, Sakha 94, and Sohag 5) and two foreign cultivars (Russian and Ukrainian), using a combination of genotyping approaches. These approaches include protein profiling by SDS-PAGE and the utilization of SCoT markers and *rbc*L chloroplast DNA barcoding.

SDS-PAGE analysis has been widely used to characterize wheat cultivars based on protein patterns [53, 54]. In this study, SDS-PAGE analysis of nine wheat cultivars revealed substantial genetic variation. A total of 44 polypeptides were identified, including 34 polymorphic and 10 monomorphic bands, resulting in a high polymorphism rate of 77.27%. Eight unique protein bands were observed, indicating distinct genetic characteristics, while the 10 monomorphic bands suggest conserved protein regions. This high level of polymorphism aligns with previous studies demonstrating the effectiveness of SDS-PAGE in differentiating wheat cultivars, such as the identification of 22 protein bands in durum wheat [55], distinct banding patterns in wheat leaf proteins [56], and the characterization of 16 polypeptides (9 polymorphic, 7 monomorphic) in durum and bread wheat endosperm [57]. These findings confirm the utility of SDS-PAGE in revealing genetic diversity based on molecular weight [58–60], making it a valuable tool for breeding programs aimed at improving nutritional quality or disease resistance.

To fully leverage the potential of protein markers in wheat breeding, integrating them with other molecular markers, such as SCoT markers, is crucial. This integrated approach can provide a more comprehensive genetic profile of cultivars, enhancing marker assisted selection, as demonstrated in various crops, including wheat [61, 62].

SCoT markers have been widely used to assess genetic diversity in *Triticum* species. In this study, 12 SCoT primers generated 148 markers with a high average polymorphism rate of 70.96% (13.2 markers per primer). This higher polymorphism rate contrasts with lower rates (8–57%) reported in *T. aestivum* cultivars from North Africa [30, 31], possibly reflecting lower genetic variation within those specific populations. Despite variations in polymorphism levels, SCoT markers have consistently demonstrated high discriminatory power among cultivars, effectively identifying specific genetic loci [30, 31].

Both SDS-PAGE analysis of protein banding profiles and SCoT marker data effectively differentiated the studied wheat cultivars. Despite targeting different molecular levels (protein expression and DNA sequences, respectively) [63], both methods revealed partially congruent patterns of genetic relatedness among the cultivars.

Cluster analysis based on SDS-PAGE data grouped the cultivars into two main clusters: one containing six Egyptian cultivars (Benisuif 6, Sakha 94, Misr 2, Misr 1, Giza 168, and Sohag 5) and another containing Gemmiza 9, Ukrainian, and Russian wheat. In contrast, SCoT marker analysis resulted in a different grouping: one group with seven bread wheat cultivars (Misr 2, Giza 168, Misr 1, Gemmiza 9, Ukrainian, Russian, and Sakha 94) and another with the two durum wheat cultivars, Benisuif 6 and Sohag 5.

The consistent clustering of Benisuif 6 and Sohag 5 in both analyses suggests a close genetic relationship, potentially attributed to shared ancestry, common traits, or similar environmental adaptations. Combined analysis of SDS-PAGE and SCoT data further refined the clustering, separating Benisuif 6 and Sohag 5 into one group and the remaining seven cultivars into another. This latter group was further subdivided into two subgroups: (1) Sakha 94, Russian, and Ukrainian; and (2) Gemmiza 9, Giza 168, Misr 1, and Misr 2, confirming the close relationships between Misr 1 and Misr 2, Russian and Ukrainian, and Benisuif 6 and Sohag 5.

Analyzing *rbc*L gene sequences is valuable for understanding genetic diversity, particularly at the interspecific level [29]. Indeed, plastid markers like *mat*K and *rbc*L have proven effective as DNA barcodes for differentiating distinct wheat landraces and related Poaceae species [64]. However, a limitation observed in this study, consistent with prior research, is that chloroplast genes such as *rbc*L often exhibit limited resolution for differentiating closely related cultivars. This is because they primarily reflect variation at higher taxonomic levels and are less sensitive to the subtle intraspecific genetic differences crucial for cultivar identification [29, 65]. Therefore, while plastid markers like *rbc*L serve as robust tools for species level discrimination, cultivar level studies ideally benefit from their combination with more variable nuclear markers to achieve comprehensive genetic differentiation and capture the full spectrum of diversity.

In this study, a notable difference in clustering patterns emerged when comparing the *rbc*L-based analysis with the combined SDS-PAGE and SCoT data. Specifically, protein profiles using SDS-PAGE and SCoT amplified allels demonstrated superior resolution in differentiating the wheat at the cultivar level compared to the chloroplast derived *rbc*L sequences. This observation aligns with established principles in molecular genetics; co-dominant molecular markers (like SCoT) target coding and non-coding regions, are often more polymorphic due to higher mutation rates and biparental inheritance. Hereby, these marker types were able to capture subtle intraspecific variations crucial for distinguishing closely related cultivars. Conversely, chloroplast genes (like *rbc*L in the present manuscript) are highly conserved, primarily reflecting broader evolutionary relationships at the species or genus level, and thus offer limited discriminatory power at the cultivar level. These findings agreed with Abouseada et al. [29] showing that SCoT markers provided higher differentiation among wheat cultivars than plastid markers like *rbc*L for intraspecific studies [29].

## Conclusions

This study successfully differentiated and characterized nine wheat cultivars of diverse origins using a combination of biophysical, biochemical, and molecular analyses, including grain morphology, biochemical composition (moisture, ash, gluten, falling number, carbohydrates, protein, minerals, and vitamins), SDS-PAGE protein profiling, SCoT markers, and *rbcL* chloroplast DNA barcoding. Grain dimensions, biochemical properties, and molecular markers all revealed significant variations among the cultivars, providing valuable information for cultivar identification and differentiation. Some Egyptian cultivars, such as Sohag 5 and Misr 1, exhibited promising physical, quality, and nutritional traits, including desirable grain weight, falling number, strong gluten content, and beneficial levels of carbohydrates, protein, and essential minerals. Protein profiling and SCoT analyses were particularly effective in revealing genetic relationships and differentiation among the studied wheat cultivars, demonstrating higher resolution than *rbcL* gene sequencing, which is more suited for higher level taxonomic comparisons. The combined use of these diverse approaches provided a comprehensive assessment of genetic variability, offering valuable insights for selecting parental lines in future breeding programs aimed at developing cultivars with improved adaptation to diverse climatic conditions and enhanced nutritional and quality traits. Future research could incorporate additional molecular markers, such as combined nuclear ribosomal internal transcribed spacer (ITS) and chloroplast DNA using specific Short Tandom Repeats (STRs) and/or barcoding (e.g., *psbA*,* trnH*, or *ndhF*) sequences, for even more refined genetic analyses.

## Materials and methods

### Plant material

This study investigated nine wheat cultivars. Seven Egyptian cultivars (Benisuif 6, Gemmiza 9, Giza 168, Misr 1, Misr 2, Sakha 94, and Sohag 5) were obtained from the Agricultural Research Center (ARC), Giza, Egypt. Two additional cultivars were imported from Russia and Ukraine by GLOBAL MISR Company. All necessary agreements and permissions were obtained, adhering to the International Union for Conservation of Nature (IUCN) guidelines established at the 27th meeting of the IUCN Council, GLAND SWITZERLAND (1989). The cultivars were identified and verified by Dr. Mohamed Tantawy, professor of plant taxonomy and flora at Ain Shams University, Cairo, Egypt. Voucher specimens are deposited in the Herbarium of the Department of Botany, Ain Shams University (CAIA; http://sweetgum.nybg.org/science/ih/herbarium-details/?irn=123925). Details on the country of origin, country code, and pedigree for each cultivar are listed in Table 3.

### Physical analyses

To assess grain quality, several parameters were measured. Grain dimensions (length, width, and thickness) were determined using a micrometer on ten randomly selected grains per cultivar, following standard procedures demonstrated by Soliman et al. [5] and Iqbal et al. [11]. Thousand grain weight was measured using a grain counter and digital balance as shown by Soliman et al. [5] and Iqbal et al. [11]. Purity and impurity percentages were calculated by manually cleaning 50 g of wheat kernels and weighing the cleaned grains and impurities. Moisture content was determined using ICC Standard Method No. 110/1 [7]. The falling number test was conducted to assess enzyme activity in flour, following the Hagberg method [66, 67]. Ash content and gluten content were determined using AACC Method 38-12.02 [7]. Whole wheat flour (100% extraction) was milled to analyze the purity of the entire grain, including the endosperm and outer layers according to Hussein et al. [68].

### Biochemical and molecular data analyses

Total carbohydrate content was determined using the method described by Prud’homme et al. [69]. Glucose and sucrose were quantified separately using the methods of Blakeney and Matheson [70] and Hubbard et al. [71], respectively. Polysaccharide content was estimated following the protocol outlined by Shabana [72]. Starch content was measured using the anthrone method according to Blakeney and Mutton [70].

### Extraction and Estimation of minerals content

Plant samples were digested using a nitric-sulfuric acid mixture, following standard procedures of Chapman and Pratt [73] and El-Beltagi et al. [74]. The digested samples were analyzed using inductively coupled plasma atomic emission spectrometry (ICP-AES) to determine the concentrations of calcium (Ca), copper (Cu), iron (Fe), magnesium (Mg), phosphorus (P), and zinc (Zn). Selenium (Se), due to its low concentration and sensitivity limitations of ICP-AES, was analyzed using graphite furnace atomic absorption spectrometry (GF-AAS) [75].

### Extraction and Estimation of vitamins content

Vitamin B1 (thiamine), B3 (niacin), B6 (pyridoxine), and folic acid were analyzed using high performance liquid chromatography (HPLC). A validated HPLC method was employed, adapted from Amidžić et al. [76]. Standard solutions of each vitamin were prepared and used for calibration. Samples were prepared by grinding and extracting with methanol, followed by filtration. Chromatographic separation was achieved using a reverse-phase C18 column and a mobile phase consisting of methanol and an aqueous buffer. Detection was performed at 290 nm.

### Extraction of total proteins (TPs) from wheat grains using SDS -PAGE technique

Wheat grain TPs were extracted following the method described by Hassanein et al. [77]. The extracted TP supernatant was stored at−-80°C for subsequent analysis. Protein concentration was determined using the Bradford method [78]. Extracted TPs were subjected to 12% SDS-PAGE according to Laemmli [32] and fully described by Hassanein et al. [77].

The protein extraction was performed on three biological replicates and three technical replicates per cultivar, as described by Hassanein et al. [77]. Protein samples were quantified and loaded onto SDS-PAGE gel, with 70 µg of each sample (equivalent to total protein content) added to 1 X of loading buffer (3:1) according to Laemmli [32] and 10 µl of protein marker at left-hand side or both sides of loaded gels. Protein concentration was determined using a BSA standard curve.

### Genomic DNA extraction

Genomic DNA was extracted by using the DNeasy Plant Mini Kit (QIAGEN, Hilden, Germany) as previously described by Abouseada et al. [29]. Quantification of DNA concentration was performed according to the Molecular Cloning Laboratory Manual [79]. DNA purity was checked by using ND-1000 spectrophotometer (Nano-Drop Technologies, Thermo Fisher Scientific Inc.).

### SCoT primers and SCoT-PCR amplification

For DNA fingerprinting, twelve SCoT primers were synthesized by HVD Egypt under license from HVD Vertriebs-Ges. GmbH (Vienna, Austria). Primers were received, rehydrated, and stored at -80 °C. SCoT-PCR was executed following the protocol described by Badr et al. [80] and Abouseada et al. [29]. The PCR amplification process was performed as follows: The initial denaturation cycle is 5 min at 94°C, followed by 40 cycles (94°C for 30 s, 50°C for 1 min, and 72°C for 2 min), with a final extension at 72°C for 7 min and then stored at 4 °C. Table 1 provides a list of SCoT primers, including their names, sequences, GC content, melting temperatures (Tm), and the number of amplicons generated per primer.

### DNA barcoding of RbcL Chloroplast gene

*rbc*L specific primer sequences (forward and reverse) used for DNA barcoding technique are given in Table 2. The PCR amplification was executed as discribed by Abouseada et al. [29]. Electrophoresis, staining, visualization, and documentation of *rbc*L specific amplicons was performed following Maniatis et al. [79]. PCR-specific amplified fragment of *rbc*L was purified from agarose gel following Abouseada et al. [29]. Cloning of the purified amplicons and the DNA sequencing protocol for the *rbc*L partial fragments were then executed as previously described by Badr et al. [80] and Abouseada et al. [29].

### Biochemical and molecular data analyses

Firstly, biochemical Analyses (biochemical, vitamin, and mineral) were conducted on ground grain powder from three biological replicates, each consisting of ten plants. Within each biological replicate, three technical replicates were analyzed. Data were analyzed using ANOVA followed by Duncan’s Multiple Range Test according to Snedecor and Cochran [81] to determine significant differences between treatment means. All statistical analyses were performed using [SPSS Statistics]. Secondly, molecular analyses were performed using three biological replicates and thre technical replicates. DNA was extracted from the three technical replicates independently. Extracted DNA from the three technical replicates was pooled in one sample for further use in both SCoT-PCR and *rbc*L amplification. In case of protein profiling the protein bands in SDS-PAGE gels were scored as “1” (present) or “0” (absent). Polymorphic metrics, including the Total Number of Alleles (TNAs), Monomorphic Alleles (MAs), and Polymorphic Alleles (PAs), along with other parameters like the percentage of polymorphism (%P), were calculated to assess the effectiveness of primers in differentiating genotypes.

While, DNA sequence analysis (*rbc*L partial sequences) were analyzed using Bayesian inference in MrBayes v3.2 [82] with the SYM + G substitution model based on the Akaike Information Criterion using MrModel-test v2.3 [83]. Phylogenetic analysis was performed using R software [84] supplemented with the “seqinr” [85], “adegenet” [86], “DECIPHER” [87], and “ape” [88] packages.

## Supplementary Information

Below is the link to the electronic supplementary material.

Supplementary Material 1

## Data Availability

The rbcL datasets generated and/or analysed during the current study are available and were deposited under indicated accession numbers (listed in the main text in Table 3) at the persistent web link approved by NCBI GenBank (https://www.ncbi.nlm.nih.gov/nuccore/). For example, PP297465.1 *T. aestivum* *rbc*L sequence could be retrieved using the following persistent web link (https://www.ncbi.nlm.nih.gov/nuccore/PP297465.1).
