# Supplementary material for: Analytical assessment of physical characteristics, metabolic processes, and molecular investigations of selected wheat (Triticum spp.) cultivars
Source: BMC Plant Biol. 2025 Aug 12;25:1067. doi: 10.1186/s12870-025-07134-0 (PMC12341216; doi:10.1186/s12870-025-07134-0)
Supplement: Supplementary file 1 — Supplementary Material 1 [file 12870_2025_7134_MOESM1_ESM.docx]

**Supplementary Tables and Figures**

**Analytical Assessment of Physical Characteristics, Metabolic Processes, and Molecular Investigations of Selected Wheat (Triticum spp.) Cultivars**

Dina H. M. Shahata ^1^, Mohamed M. El-Mahdy*^2^*, Mohamed Ibrahim^1^*, Magda M. I. EL Araby ^1^, Somia S. El -Akkad ^1^ , Faten Y. Ellmouni^3^*

^1^ Department of Botany, Faculty of Science, Ain Shams University, Cairo, Egypt

^2^ Agricultural Genetic Engineering Research Institute, Agricultural Research Centre (ARC), Giza, Egypt

^3^ Botany Department, Faculty of Science, Fayoum University, Fayoum 63514, Egypt

*Correspondence: [fyl00@fayoum.edu.eg](mailto:fyl00@fayoum.edu.eg) (F.Y.E.) ORCID no. 0000-0002-9463-0008;

[m.shehata@sci.asu.edu.eg](mailto:m.shehata@sci.asu.edu.eg) (M.I.) ORCID no. 0000-0002-5401-5115

**Supplementary Table S1** Grain dimensions of nine wheat cultivars. The cultivars include seven Egyptian cultivars (Benisuif 6, Gemmiza 9, Giza 168, Misr 1, Misr 2, Sakha 94, and Sohag 5) and two foreign cultivars (Russian and Ukrainian). Data represent the mean of ten replicates with standard deviation (SD) included.

| **Cultivars** | **Width (mm)** | **Thickness (mm)** | **Length (mm)** |
| --- | --- | --- | --- |
| Giza 168 | 2.52 ± 0.14^ab^ | 1.94 ± 0.04^c^ | 5.36 ± 0.06^e^ |
| Sohag 5 | 2.48 ± 0.06^ab^ | 2.21 ± 0.05^b^ | 6.40 ± 0.14^b^ |
| Gemmiza 9 | 2.40 ± 0.12^bc^ | 1.9 ± 0.12^c^ | 6.11± 0.19^c^ |
| Misr 1 | 2.41 ± 0.17^bc^ | 1.98 ± 0.17^c^ | 5.75 ± 0.19^d^ |
| Misr 2 | 2.61 ± 0.14^a^ | 1.98 ± 0.09^c^ | 5.71 ± 0.22^d^ |
| Sakha 94 | 2.31 ± 0.15^c^ | 1.92 ± 0.06^c^ | 5.09 ± 0.18^f^ |
| Benisuif 6 | 2.51 ± 0.16^ab^ | 2.34 ± 0.08^a^ | 7.32 ± 0.15^a^ |
| Russian | 1.89 ± 0.26^d^ | 1.85 ± 0.19^c^ | 5.83 ± 0.38^d^ |
| Ukrainian | 1.85 ± 0.26^d^ | 1.91 ± 0.09^c^ | 5.90 ± 0.23^d^ |

**Supplementary Table S2** Physical analyses of grains and whole wheat flour of nine wheat cultivars. The cultivars include seven Egyptian cultivars (Benisuif 6, Gemmiza 9, Giza 168, Misr 1, Misr 2, Sakha 94, and Sohag 5) and two foreign cultivars (Russian and Ukrainian). Data represent the meaning of three replicates with standard deviation (SD) included.

| Cultivars | Thousand Grain Weight (Gram) | Moisture (%) | Ash Content (%) | Gluten (%) | Dry Gluten (%) | Gluten Index (%) |
| --- | --- | --- | --- | --- | --- | --- |
| Giza 168 | 44.07 ± 0.15^c^ | 11.87 ± 0.42^c^ | 1.49 ± 0.16^bcd^ | 28.30 ± 0.30^b^ | 9.35 ± 0.15^de^ | 28.22 ± 2.53^d^ |
| Sohag 5 | 50.37 ± 0.65^b^ | 12.37 ± 0.49^b^ | 1.67 ± 0.08^a^ | 26.30 ± 0.10^cd^ | 12.90 ± 0.30^a^ | 92.17 ± 0.15^a^ |
| Gemmiza 9 | 58.50 ± 1.50^a^ | 12.53 ± 0.32^b^ | 1.63 ± 0.09^ab^ | 29.50 ± 0.30^a^ | 9.10 ± 0.10^e^ | 64.47 ± 12.05^c^ |
| Misr 1 | 44.40 ± 0.90^c^ | 10.60 ± 0.36^d^ | 1.5 ± 0.03^bcd^ | 29.80 ± 0.60^a^ | 9.75 ± 0.25^cde^ | 83.00 ± 5.70^b^ |
| Misr 2 | 44.37 ± 0.95^c^ | 10.87 ± 0.21^d^ | 1.46 ± 0.04^cd^ | 26.80 ± 0.40^cd^ | 8.75 ± 0.45^e^ | 75.90 ± 1.70^b^ |
| Sakha 94 | 45.50 ± 1.20^c^ | 13.30 ± 0.10^a^ | 1.38 ± 0.04^d^ | 27.07 ± 1.05^c^ | 10.97 ± 1.65^bc^ | 74.80 ± 0.60^b^ |
| Benisuif 6 | 58.77 ± 1.25^a^ | 12.40 ± 0.00^b^ | 1.43 ± 0.05^cd^ | 26.00 ± 0.70^d^ | 10.70 ± 0.30^bcd^ | 56.70 ± 2.89^c^ |
| Russian | 36.90 ± 0.10^e^ | 10.93 ± 0.06^d^ | 1.55 ± 0.05^abc^ | 22.30 ± 0.10^f^ | 9.47 ± 0.75^de^ | 95.50 ± 0.29^a^ |
| Ukrainian | 40.57 ± 0.85^d^ | 11.47 ± 0.06^d^ | 1.55 ± 0.01^abc^ | 24.97 ± 0.25^e^ | 11.40± 1.30^b^ | 98.20 ± 0.12^a^ |

**Supplementary Table S3** Physical analyses (Purity%, Impurity% and Fallen number) of grains and whole wheat flour of nine wheat cultivars. The cultivars include seven Egyptian cultivars (Benisuif 6, Gemmiza 9, Giza 168, Misr 1, Misr 2, Sakha 94, and Sohag 5) and two foreign cultivars (Russian and Ukrainian).

| Cultivars | Purity (%) | Impurity (%) | Falling number (sec.) |
| --- | --- | --- | --- |
| Giza 168 | 98.8 | 1.20 | 339 |
| Sohag 5 | 99.04 | 0.96 | 413 |
| Gemmiza 9 | 99.90 | 0.10 | 344 |
| Misr 1 | 97.60 | 2.40 | 309 |
| Misr 2 | 97.30 | 2.70 | 381 |
| Sakha 94 | 99.48 | 0.52 | 165 |
| Benisuif 6 | 99.30 | 0.70 | 366 |
| Russian | 97.50 | 2.50 | 402 |
| Ukrainian | 98.80 | 1.20 | 308 |

**Supplementary Table S4** Biochemical analyses of whole wheat flour of nine wheat cultivars. The cultivars include seven Egyptian cultivars (Benisuif 6, Gemmiza 9, Giza 168, Misr 1, Misr 2, Sakha 94, and Sohag 5) and two foreign cultivars (Russian and Ukrainian). The table shows the content of total carbohydrates (reducing sugars, non-reducing sugars, polysaccharide and starch) and total soluble protein. Values represent the meaning of four replicates with standard deviation (SD) included.

| Cultivars | Reducing sugar (mg/g) | Non-reducing sugar (mg/g) | Polysaccharide (mg/g) | Starch (mg/g) | Total carbohydrate (mg/g) | Protein (mg/g) |
| --- | --- | --- | --- | --- | --- | --- |
| Giza 168 | 10.31 ± 1.25^b^ | 1.94 ± 0.07^bc^ | 262.03 ± 1.20^a^ | 476.91 ± 14.09^b^ | 751.19 | 13.90 ± 0.75^a^ |
| Sohag 5 | 7.43 ± 0.66^de^ | 1.86 ± 0.01^d^ | 134.07 ± 3.32^f^ | 476.91 ± 10.89^b^ | 620.27 | 13.00 ± 0.29^ab^ |
| Gemmiza 9 | 8.38 ± 0.61^cd^ | 2.00 ± 0.05^b^ | 239.55 ± 22.12^b^ | 430.87 ± 17.59^c^ | 680.8 | 14.40 ± 0.31^a^ |
| Misr 1 | 11.76 ± 0.88^a^ | 2.47 ± 0.02^a^ | 149.57 ± 6.61^e^ | 534.53 ± 71.85^a^ | 698.33 | 11.94 ± 0.98^b^ |
| Misr 2 | 8.72 ± 0.82^c^ | 2.41 ± 0.03^a^ | 128.97 ± 4.82^f^ | 474.65 ± 15.85^b^ | 614.75 | 11.83 ± 0.74^b^ |
| Sakha 94 | 9.99 ± 0.71^b^ | 1.84 ± .01^d^ | 193.20 ± 4.97^d^ | 427.66 ± 1.61^c^ | 632.69 | 13.63 ± 2.54^a^ |
| Benisuif 6 | 7.35 ± 1.00^de^ | 1.65 ± 0.05^e^ | 77.66 ± 4.64^g^ | 461.94 ± 0.64^bc^ | 548.6 | 13.13 ± 0.31^ab^ |
| Russian | 6.31 ± 0.67^e^ | 1.68 ± 0.07^e^ | 222.98 ± 7.99^c^ | 421.38 ± 10.99^c^ | 652.35 | 14.39 ± 1.14^a^ |
| Ukrainian | 4.60 ± 0.06^f^ | 1.89 ± 0.04^cd^ | 68.41 ± 0.72^g^ | 526.96 ± 3.58^a^ | 601.86 | 14.14 ± 0.25^a^ |

**Supplementary Table S5** Minerals content (mg/g) of whole wheat flour of nine wheat cultivars (seven Egyptian cultivars; Benisuif 6, Gemmiza 9, Giza 168, Misr 1, Misr 2, Sakha 94, and Sohag 5) and two foreign cultivars; Russian and Ukrainian).

| Cultivars | Minerals (mg/g) | | | | | | |
| --- | --- | --- | --- | --- | --- | --- | --- |
|  | Mg | Cu | Fe | P | Zn | Ca | Se |
| Benisuif 6 | 2.25 | 0.12 | 1.01 | 0.0075 | 1.54 | 20.88 | 0.0011 |
| Gemmiza 9 | 2.63 | 0.08 | 0.08 | 0.0030 | 1.14 | 26.87 | 0.0011 |
| Giza 168 | 1.88 | 0.08 | 0.13 | 0.0055 | 1.55 | 14.99 | 0.0832 |
| Misr 1 | 1.88 | 0.09 | 0.23 | 0.0075 | 1.59 | 21.57 | 0.0011 |
| Misr 2 | 2.00 | 0.15 | 0.06 | 0.0080 | 1.65 | 20.25 | 0.0011 |
| Sakha 94 | 2.13 | 0.18 | 0.10 | 0.0010 | 1.38 | 18.08 | 0.0220 |
| Sohag 5 | 2.75 | 0.06 | 0.14 | 0.0040 | 1.72 | 22.04 | 0.0700 |
| Russian | 2.63 | 0.09 | 1.24 | 0.0040 | 1.19 | 20.67 | 0.0011 |
| Ukrainian | 1.75 | 0.06 | 0.11 | 0.0095 | 1.12 | 18.86 | 0.0220 |

**Supplementary Table S6** Vitamins content (mg/g) of whole wheat flour of nine wheat cultivars (seven Egyptian cultivars; Benisuif 6, Gemmiza 9, Giza 168, Misr 1, Misr 2, Sakha 94 and Sohag 5, and two foreign cultivars; Russian and Ukrainian).

| Cultivars | Vitamins (mg/g) | | | |
| --- | --- | --- | --- | --- |
|  | Thiamine (B1) | Niacin (B3) | Pyridoxine (B6) | Folic acid (B9) |
| Benisuif 6 | 0.0208 | 0.8850 | 0.3488 | 0.7041 |
| Gemmiza 9 | 0.0035 | 1.4821 | 0.5547 | 0.2971 |
| Giza 168 | 0.0011 | 1.5330 | 0.3194 | 0.1625 |
| Misr 1 | 0.0025 | 0.2764 | 0.0929 | 0.0387 |
| Misr 2 | 0.0009 | 0.2394 | 0.2356 | 0.0002 |
| Sakha 94 | 0.0015 | 1.3388 | 0.6544 | 0.5573 |
| Sohag 5 | 0.0058 | 0.8182 | 0.2420 | 0.2692 |
| Russian | 0.0190 | 0.1130 | 0.0530 | 0.1520 |
| Ukrainian | 0.0315 | 0.1543 | 0.4883 | 0.0435 |

**Supplementary Table S7** Gel analysis of total proteins (TPs) in nine wheat cultivars (seven Egyptian cultivars; Giza 168, Sohag 5, Gemmiza 9, Misr 1, Misr 2, Sakha 94, Benisuif 6, and two foreign cultivars; Russian and Ukrainian)

| Row no. | Cultivars | | | | | | | | | Frequency | Polymorphism |
| --- | --- | --- | --- | --- | --- | --- | --- | --- | --- | --- | --- |
|  | **Giza 168** | **Sohag 5** | **Gemmiza 9** | **Misr 1** | **Misr 2** | **Sakha 94** | **Benisuif 6** | **Russian** | **Ukrainian** |  |  |
|  | 1 | 2 | 3 | 4 | 5 | 6 | 7 | 8 | 9 |  |  |
| 1 | 1 | 0 | 1 | 1 | 1 | 1 | 0 | 1 | 1 | 0.78 | Polymorphic |
| 2 | 1 | 1 | 1 | 1 | 1 | 1 | 1 | 1 | 0 | 0.89 | Polymorphic -U (NUB) |
| 3 | 1 | 1 | 1 | 1 | 1 | 1 | 1 | 1 | 1 | 1 | Monomorphic |
| 4 | 0 | 1 | 0 | 0 | 0 | 0 | 1 | 1 | 1 | 0.44 | Polymorphic |
| 5 | 1 | 0 | 1 | 1 | 1 | 1 | 0 | 0 | 1 | 0.67 | Polymorphic |
| 6 | 1 | 1 | 1 | 1 | 1 | 1 | 1 | 1 | 0 | 0.89 | Polymorphic -U (NUB) |
| 7 | 1 | 1 | 1 | 1 | 1 | 1 | 1 | 1 | 1 | 1 | Monomorphic |
| 8 | 1 | 1 | 1 | 1 | 1 | 1 | 1 | 1 | 1 | 1 | Monomorphic |
| 9 | 1 | 1 | 1 | 1 | 1 | 1 | 1 | 1 | 1 | 1 | Monomorphic |
| 10 | 1 | 1 | 1 | 1 | 1 | 1 | 1 | 1 | 1 | 1 | Monomorphic |
| 11 | 1 | 1 | 1 | 1 | 1 | 1 | 1 | 1 | 1 | 1 | Monomorphic |
| 12 | 1 | 1 | 1 | 1 | 1 | 1 | 1 | 1 | 1 | 1 | Monomorphic |
| 13 | 1 | 1 | 1 | 1 | 1 | 1 | 1 | 0 | 0 | 0.78 | Polymorphic |
| 14 | 1 | 1 | 1 | 1 | 1 | 1 | 0 | 1 | 1 | 0.89 | Polymorphic -U (NUB) |
| 15 | 1 | 0 | 1 | 0 | 0 | 1 | 0 | 1 | 1 | 0.56 | Polymorphic |
| 16 | 1 | 1 | 1 | 1 | 1 | 1 | 1 | 1 | 1 | 1 | Monomorphic |
| 17 | 0 | 1 | 0 | 0 | 0 | 0 | 1 | 0 | 0 | 0.22 | Polymorphic |
| 18 | 1 | 1 | 1 | 1 | 1 | 1 | 1 | 1 | 1 | 1 | Monomorphic |
| 19 | 1 | 0 | 0 | 1 | 1 | 0 | 1 | 1 | 1 | 0.67 | Polymorphic |
| 20 | 0 | 1 | 0 | 1 | 1 | 0 | 1 | 0 | 0 | 0.44 | Polymorphic |
| 21 | 0 | 1 | 0 | 0 | 0 | 0 | 1 | 1 | 1 | 0.44 | Polymorphic |
| 22 | 1 | 0 | 1 | 1 | 1 | 1 | 0 | 0 | 1 | 0.67 | Polymorphic |
| 23 | 0 | 0 | 1 | 1 | 0 | 0 | 0 | 1 | 0 | 0.33 | Polymorphic |
| 24 | 1 | 1 | 1 | 1 | 0 | 1 | 1 | 1 | 1 | 0.89 | Polymorphic -U (NUB) |
| 25 | 1 | 0 | 1 | 1 | 1 | 1 | 1 | 1 | 1 | 0.89 | Polymorphic -U (NUB) |
| 26 | 0 | 1 | 1 | 1 | 1 | 1 | 1 | 0 | 1 | 0.78 | Polymorphic |
| 27 | 1 | 1 | 1 | 1 | 1 | 1 | 0 | 1 | 0 | 0.78 | Polymorphic |
| 28 | 0 | 0 | 0 | 0 | 0 | 1 | 1 | 1 | 1 | 0.44 | Polymorphic |
| 29 | 1 | 1 | 1 | 1 | 1 | 1 | 1 | 0 | 0 | 0.78 | Polymorphic |
| 30 | 0 | 0 | 1 | 1 | 1 | 1 | 0 | 1 | 1 | 0.67 | Polymorphic |
| 31 | 1 | 1 | 0 | 1 | 1 | 0 | 1 | 0 | 1 | 0.56 | Polymorphic |
| 32 | 1 | 1 | 1 | 1 | 1 | 1 | 0 | 1 | 0 | 0.78 | Polymorphic |
| 33 | 0 | 0 | 1 | 0 | 0 | 0 | 0 | 0 | 1 | 0.22 | Polymorphic |
| 34 | 1 | 0 | 1 | 1 | 1 | 1 | 1 | 1 | 1 | 0.89 | Polymorphic -U (NUB) |
| 35 | 1 | 1 | 0 | 1 | 1 | 1 | 1 | 1 | 1 | 0.89 | Polymorphic -U (NUB) |
| 36 | 0 | 1 | 1 | 0 | 0 | 1 | 0 | 0 | 0 | 0.33 | Polymorphic |
| 37 | 1 | 1 | 1 | 1 | 1 | 0 | 1 | 1 | 1 | 0.89 | Polymorphic -U (NUB) |
| 38 | 1 | 1 | 0 | 0 | 0 | 0 | 0 | 0 | 0 | 0.22 | Polymorphic |
| 39 | 0 | 0 | 0 | 0 | 1 | 1 | 1 | 0 | 1 | 0.44 | Polymorphic |
| 40 | 1 | 1 | 1 | 1 | 0 | 0 | 0 | 1 | 0 | 0.56 | Polymorphic |
| 41 | 0 | 0 | 0 | 0 | 1 | 1 | 1 | 1 | 1 | 0.56 | Polymorphic |
| 42 | 1 | 1 | 1 | 1 | 0 | 1 | 1 | 0 | 0 | 0.67 | Polymorphic |
| 43 | 1 | 1 | 1 | 1 | 1 | 1 | 1 | 1 | 1 | 1 | Monomorphic |
| 44 | 1 | 1 | 1 | 1 | 1 | 0 | 0 | 1 | 1 | 0.78 | Polymorphic |
| Monomorphic bands | | | | | | | | | | 10 |  |
| Polymorphic (without Unique) | | | | | | | | | | 26 |  |
| Unique bands | | | | | | | | | | 8 |  |
| Polymorphic (with Unique) | | | | | | | | | | 34 |  |
| Total number of bands | | | | | | | | | | 44 |  |
| Polymorphism (%) | | | | | | | | | | 77.27% |  |
| Mean of band frequency | | | | | | | | | | 0.72 |  |

**Supplementary Fig. S1** Photographs of wheat grains of the seven Egyptian cultivars (1-7) and two foreign cultivars (8-9) under stereomicroscope. 1 represents Giza 168, 2 represents Sohag 5, 3 represents Gemmiza 9, 4 represents Misr 1, 5 represents Misr 2, 6 represents Sakha 94, 7 represents Benisuif 6, 8 represents Russian and 9 represents Ukrainian.


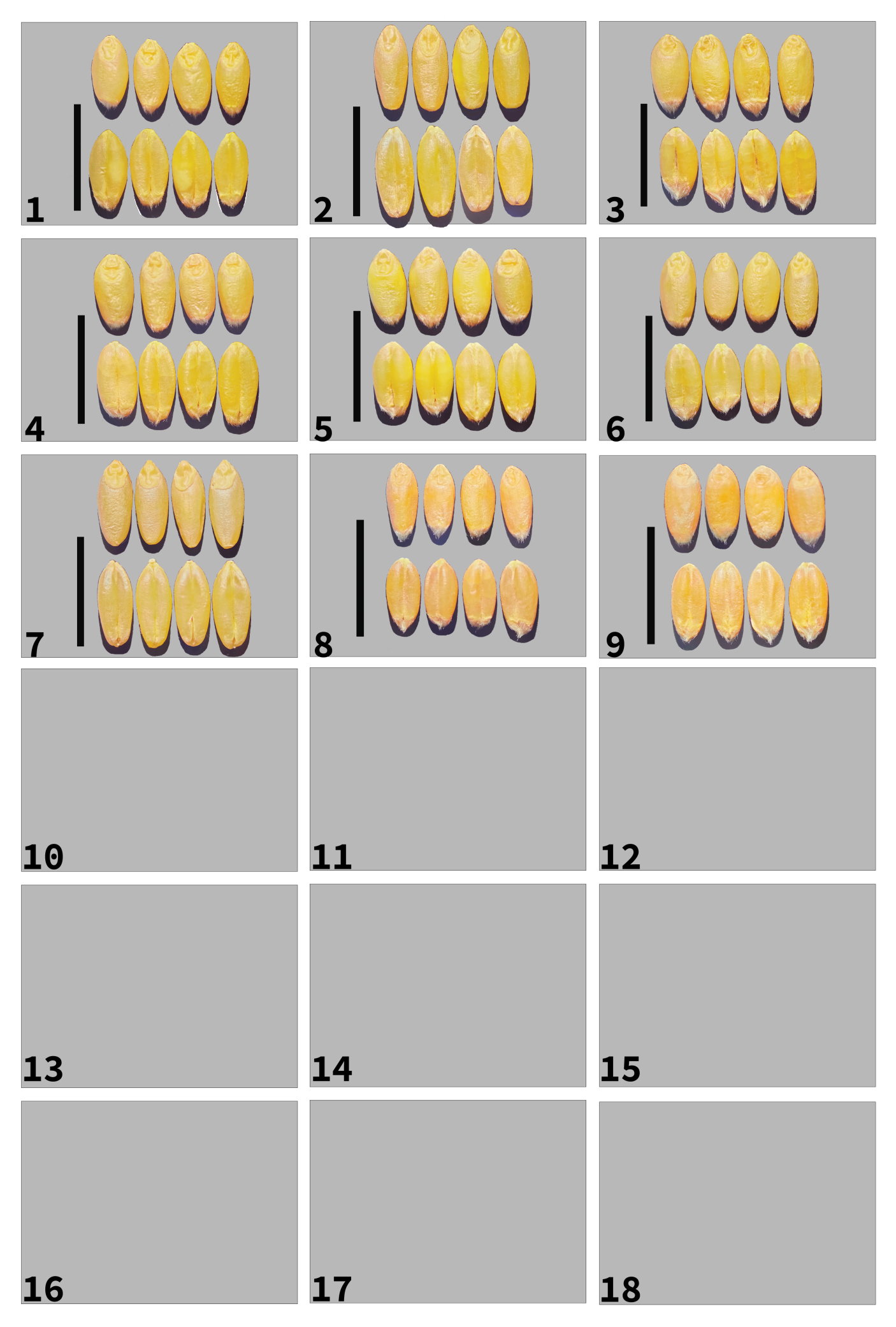


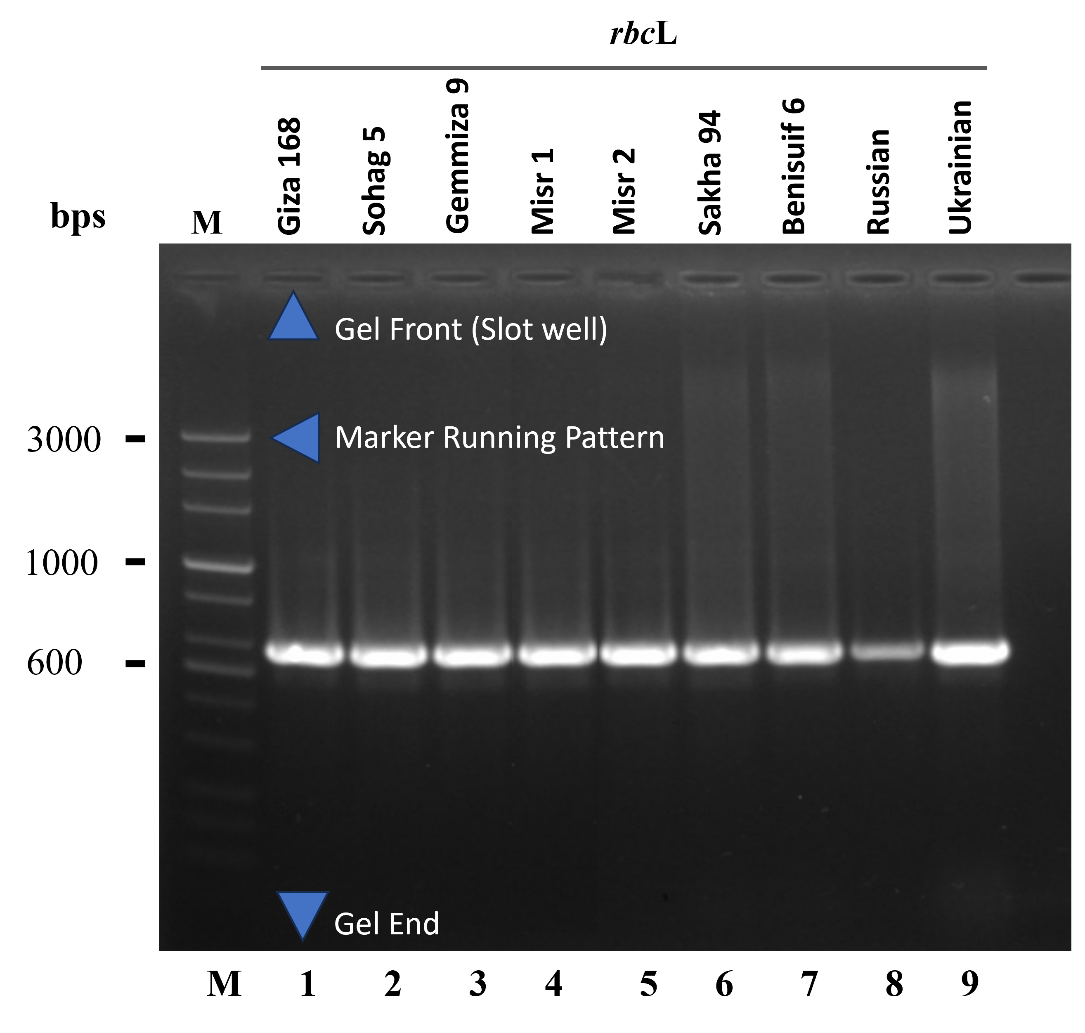
**Supplementary Fig. S2** Amplification of DNA barcoding loci of *rbc*L gene. Agarose gel electrophoresis of the specific PCR products of *rbc*L was shown. Blue arrowheads indicate the gel front (starting from the slot wells), the full running pattern of the DNA size marker, and the gel end. Also, the molecular size of DNA size marker is denoted by the numbers left-handed of the agarose gel shown. **(Chloroplast DNA barcoding fully uncropped figure**
